# Meta-Analysis of Stenting versus Non-Stenting for the Treatment of Ureteral Stones

**DOI:** 10.1371/journal.pone.0167670

**Published:** 2017-01-09

**Authors:** Hai Wang, Libo Man, Guizhong Li, Guanglin Huang, Ning Liu, Jianwei Wang

**Affiliations:** Department of Urology, Beijing Jishuitan Hospital, Beijing, China; Taipei Medical University, TAIWAN

## Abstract

**Background and aim:**

Ureteroscopic lithotripsy (URL) and extracorporeal shock wave lithotripsy (ESWL) are two widely used methods for the treatment of ureteral stones. The need for ureteral stenting during these procedures is controversial. In this meta-analysis, we evaluated the benefits and disadvantages of ureteral stents for the treatment of ureteral stones.

**Methods:**

Databases including PubMed, Embase and Cochrane library were selected for systematic review of randomized controlled trials (RCTs) comparing outcomes with or without stenting during URL and ESWL. Meta-analysis was performed using RevMan 5.3 and STATA 13.0 software.

**Results:**

We identified 22 RCTs comparing stenting and non-stenting. The stented group was associated with longer operation time (WMD: 4.93; 95% CI: 2.07 to 7.84; *p* < 0.001), lower stone-free rate (OR: 0.55; 95% CI: 0.34 to 0.89; *p* = 0.01). In terms of complications, the incidence of hematuria (OR: 3.68; 95% CI: 1.86 to 7.29; *p* < 0.001), irritative urinary symptoms (OR: 4.40; 95% CI: 2.19 to 9.10; *p* < 0.001), urinary infection (OR: 2.23; 95% CI: 1.57 to 3.19; *p* < 0.001), and dysuria (OR: 3.90; 95% CI: 2.51 to 6.07; *p* < 0.001) were significantly higher in the stented group. No significant differences in visual analogue score (VAS), stricture formation, fever, or hospital stay were found between stenting and non-stenting groups. The risk of unplanned readmissions (OR: 0.63; 95% CI: 0.41 to 0.97; *p* = 0.04) was higher in the non-stented group.

**Conclusions:**

Our analysis showed that stenting failed to improve the stone-free rate, and instead, it resulted in additional complications. However, ureteral stents are valuable in preventing unplanned re-hospitalization. Additional randomized controlled trials are still required to corroborate our findings.

## Introduction

Urolithiasis is the most common urological disease with a prevalence rate of 10–15% and a recurrence rate of 50% [1]. In some countries with a high standard of life, this rate is significantly high and has increased more than 37% over the last 20 years [2]. Ureteral stones usually result in ureteric obstruction, renal colic, infection and hydronephrosis. Ureteroscopic lithotripsy (URL), extracorporeal shock wave lithotripsy (ESWL), medical therapy, percutaneous nephrolithotomy and laparoscopic surgery are all the indicated for the treatment of ureteral stones. URL and ESWL are the most widely used techniques to clear stones with high degree of success [3]. The routine insertion of ureteral stents over a prolonged period reduces the risk of ureteral obstruction and renal colic [4]. The stents provide the path for drainage of stone fragments down to the bladder and improve hydronephrosis simultaneously. Moreover, long-term stent implantation promotes healing of mucosal injury caused by surgeries and prevents the formation of ureteral strictures [5]. However, the use of ureteral stents for the treatment of ureteral stones is still controversial, given the stent-associated complications including irritation and discomfort in addition to inherent risks of stent migration, vesico-ureteral reflux and stent encrustation [6].

In recent years, a number of studies discussed the need for ureteral stents in URL and ESWL. A few urologists suggested that ureteral stents were unnecessary before or after URL and ESWL, because of complications although stenting improved the stone-free rate [7]. And according to the current American Urological Association (AUA) guidelines, the placement of ureter stents is not required in the surgical treatment of ureteral stones [8]. Other studies suggested that routine stenting was desirable for prophylaxis [4,6]. In the absence of a definitive conclusion, we conducted a meta-analysis of published studies, to evaluate the need for ureteral stents for the treatment of ureteral stones.

## Materials and Methods

### Study selection

This meta-analysis was performed according to the PRISMA guidelines [9] (S1 Checklist). A systematic search of Pubmed, Embase and Cochrane online library was conducted to identify all the studies published through March 22, 2016 comparing stenting with non-stenting. We used MESH search headings: “ureteral calculi”, “lithotripsy”, “ureteral stent” and “randomized controlled trials”. The “related articles” function was used to broaden the search, and all the abstracts, studies, and citations were reviewed. We conducted manual searches of reference lists from the relevant original and review articles to identify additional eligible studies.

### Inclusion and exclusion criteria

The inclusion criteria were as follows: (1) randomized controlled trails (RCTs), (2) comparison of stenting and non-stenting, (3) patients with ureteral stones treated with URL or ESWL, (4) reports of at least one outcome of interest such as operation time, VAS, stone-free rate, complications, and related data.

Studies were excluded if they involved: (1) patients with stones in kidney, bladder or urethra, or other accompanying diseases, (2) no outcomes of interest (specified later) either reported, or impossible to calculate or extrapolate based on the available data.

### Data extraction and outcomes of interest

Two reviewers independently extracted the following data including: first author, year of publication, country, study interval, study design, the number of patients with and without stents, characteristics of the study population, and outcomes of interest. All the disagreements related to eligibility were resolved by a third reviewer through discussion until a consensus was reached.

The following outcomes were extracted to compare stenting and non-stenting. Baseline demographic variables included: age, proportion of males, stone size and degree of distal location. Perioperative and postoperative variables included operating time, visual analogue scale (VAS), length of hospital stay, stone-free rate and readmission, complications including pain, dysuria, urinary infection, hematuria, fever, irritative symptoms and ureteral strictures.

### Study quality

Two reviewers independently assessed the quality of trials and any disagreement was resolved by consensus. The quality of included RCTs was evaluated based on Cochrane risk of bias according to the criteria prescribed by the Cochrane Handbook for Systematic Reviews of Intervention [10]. Selection bias (random sequence generation and allocation concealment), performance bias (blinding of participants and personnel), detection bias (blinding of outcome assessment), attrition bias (incomplete outcome data), reporting bias (selective reporting), and other biases were assessed using RevMan 5.3 (Cochrane Library Software, Oxford, UK). Three potential types of bias including low risk, high risk, and unclear risk, were determined for each single trial during the assessment. A low-risk bias was suggested when all the seven items met the criteria as “low risk”, and a high risk of bias was suggested when at least one of the seven items was assessed as “high risk”.

### Statistical analysis

Our meta-analysis was performed according to the recommendations of the Cochrane Collaboration and the Quality of Reporting of Meta-analyses (QUORUM) guidelines [11]. The weighted mean difference (WMD) was used for continuous variables and the odds ratio (OR) was used for dichotomous parameters both with 95% confidence intervals (CIs). Studies presenting continuous data as means and ranges were subjected to an approximate transformation using the technique described by Hozo [12]. All the pooled effects were determined using the z test and *p* < 0.05 was considered statistically significant. The heterogeneity of the treatment effects in the included trials was evaluated using Q and I^2^ statistics. When the I^2^ value was less than 50% and *p* > 0.1, the evidence showed no significant heterogeneity, and we used fixed-effects (FE) model. Otherwise, we used random-effects (RE) model. To evaluate the potential for heterogeneity, we conducted a subgroup analysis based on the trials published within the last 10 years (published after 2006). The variables were pooled only if the outcomes were reported in three or more studies in each subgroup. We also created subgroups based on the treatment methods: USL and ESWL. Due to the limited number (three) of trials involving ESWL, the subgroup analysis for ESWL may be of limited significance. Sensitivity analyses were performed by omitting a specific study each time. All the statistical analyses were performed using RevMan 5.3 (Cochrane Library Software, Oxford, UK). We used Egger’s and Begg’s tests to assess publication bias. All the reported P values were two-sided and p values less than 0.05 were regarded as significant for all included trials. This analysis was conducted using STATA (Version 13.0; Stata Corp, Texas, United States of America).

## Results

### Characteristics of selected studies

**Fig 1:**
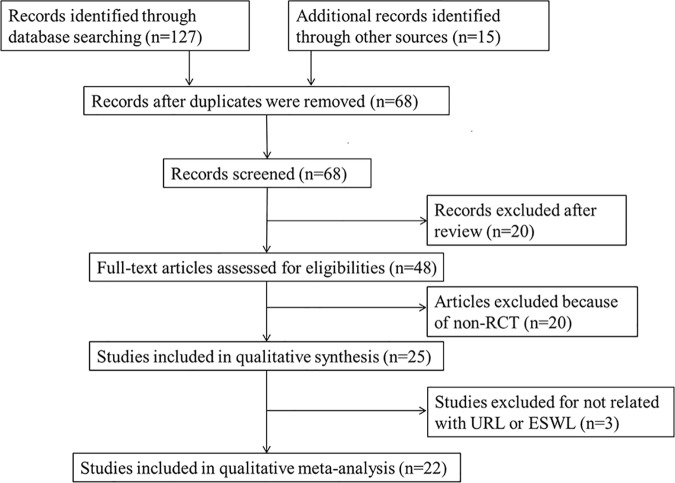
Flowchart outlining the selection of studies for meta-analysis.

**Fig 2:**
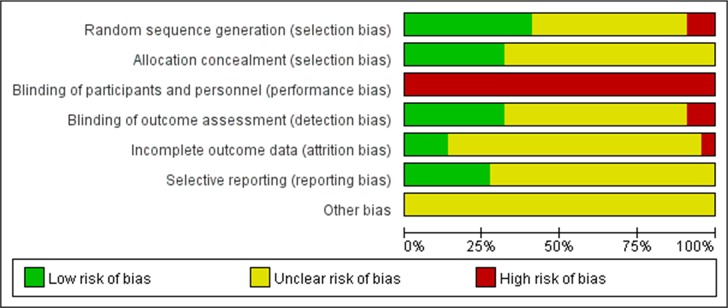
Graph indicating risk of bias in each included trial.

**Fig 3:**
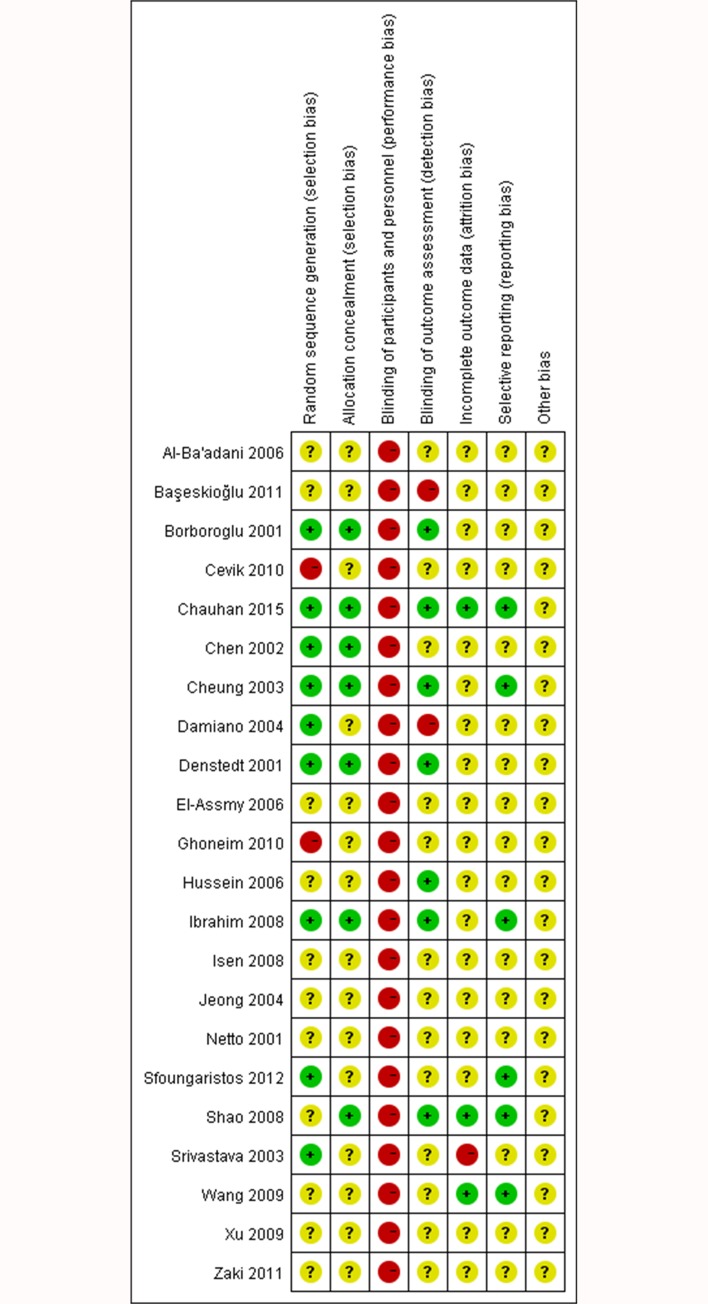
Summary of the risk of bias assessment in each included trial.

We retrieved 221 records through database search, and 22 trials [7,13–33] were selected for meta-analysis (Fig 1), including 1257 patients with ureteral stents and 1295 patients without ureteral stents, respectively. According to the criteria discussed previously, all the included trials were deemed to show a high risk of bias (Figs 2 and 3). The characteristics of these studies are shown in Table 1. The most common diameter of ureteral stents used in the trials was 6F ranging from 4.5 to 7F, although only two trials mentioned the manufacturing company. The duration of stents varied from 3 days to 6 weeks in different patients. There was no significant difference with respect to age, proportion of males, stone size or extent of lower ureteral stones in the stented and unstented groups (Table 2).

**Table 1:** Characteristics of included studies.

First author/year	Country	Study interval	Study type	Surgical approach	No. of patients, Stented/Unstented	Follow-up, months	Company/Diameter of stents	Duration of stent, days
Borboroglu 2001	USA	1998–2001	RCT	URL	53/54	1	NR/6F	3–10
Denstedt 2001	Canada	NR	RCT	URL	29/29	3	NR/NR	7
Netto 2001	Brazil	1997–2000	RCT	URL	133/162	3	NR/NR	1–3
Chen 2002	Taiwan	2000–2000	RCT	URL	30/30	1	NR/7F	3
Cheung 2003	HongKong	2001–2002	RCT	URL	29/29	3	NR/6F	14
Srivastava 2003	India	2000–2002	RCT	URL	26/22	3	NR/6F	21
Damiano 2004	Italy	2000–2002	RCT	URL	52/52	3	NR/4.5 or 6F	14
Jeong 2004	Korea	2000–2001	RCT	URL	23/22	NR	NR/7F	7
Hussein 2006	Egypt	2003–2004	RCT	URL	28/28	6	NR/6F	21
El-Assmy 2006	Egypt	2001–2004	RCT	ESWL	93/93	3	NR/6F	14–42
Al-Ba'adani 2006	Yemen	2004–2005	RCT	URL	40/45	1	NR/6F	2–28
Isen 2008	Turkey	2004–2007	RCT	URL	21/22	NR	NR/4.8F	21
Ibrahim 2008	Egypt	2004–2006	RCT	URL	110/110	24	NR/6F	14
Shao 2008	China	2005–2006	RCT	URL	58/57	3	Cook IrelandLtd/4.7F	14
Wang 2009	Taiwan	2004–2007	RCT	URL	71/67	3	NR/7F	7
Xu 2009	China	2005–2006	RCT	URL	55/55	3	NR/4.8F	21
Ghoneim 2010	Egypt	2007–2008	RCT	ESWL	30/30	3	Rusch International/6F	>7
Cevik 2010	Turkey	2005–2007	RCT	URL	30/30	3	NR/4.8F	21
Başeskioğlu 2011	Turkey	2005–2010	RCT	URL	144/142	6	NR/NR	NR
Zaki 2011	Pakistan	2008–2010	RCT	URL	99/99	3	NR/6F	5
Sfoungaristos 2012	Greece	2009–2011	RCT	ESWL	70/86	NR	NR/6F	NR
Chauhan 2015	India	2011–2014	RCT	URL	33/31	NR	NR/5F	14

**Table 2:** Clinical demographics: stenting vs. non-stenting.

Outcome of interest		No. of studies		No. of patients Stented/Unstented		OR/ WMD (95% CI)^†^		*p*-value		Study heterogeneity							
										Chi^2^		df		*I*^*2*^		*p*-value	
Age (year)		19		1021/1016		0.55 [-0.81, 1,92]^†^		0.43		27.79		18		35%		**0.07**	
Proportion of males		20		1154/1178		0.97 [0.82, 1.16]		0.77		17.15		19		0%		0.58	
Mean stone size (mm)		20		1154/1178		0.17 [-0.16, 0.50]^†^		0.32		76.45		19		75%		**<0.001**	
Proportion of lower ureteral stones		12		760/786		0.86 [0.68, 1.08]		0.19		13.9		11		21%		0.24	

CI = confidence interval; OR = odds ratio; WMD = weighted mean difference

^†^Values of WMD

Statistically significant results are shown in bold.

### Outcomes of perioperative variables

**Fig 4:**
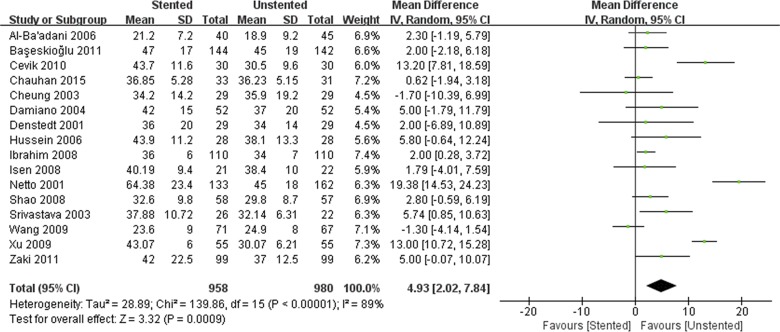
Forest plot and meta-analysis of operation time (min).

**Fig 5:**
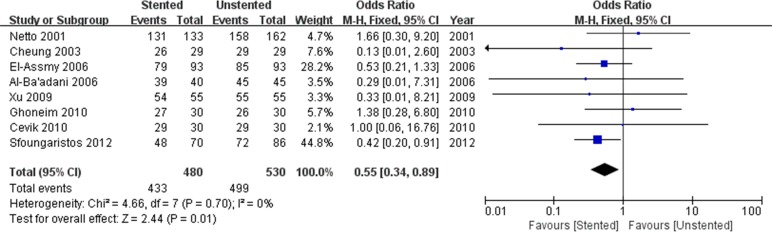
Forest plot and meta-analysis of stone-free rate.

**Fig 6:**
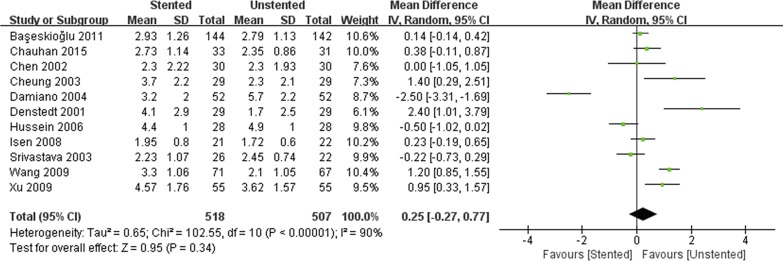
Forest plot and meta-analysis of visual analogue score (VAS).

**Fig 7:**
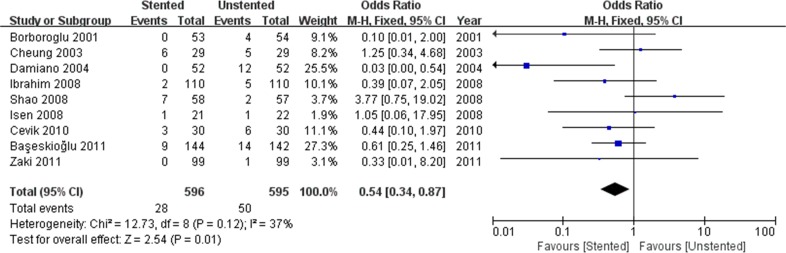
Forest plot and meta-analysis readmission rate.

The stented group was associated with longer operation time/min (WMD: 4.93; 95% CI: 2.07 to 7.84; *p* < 0.001) (Fig 4), and lower stone-free rate (OR: 0.55; 95% CI: 0.34 to 0.89; *p* = 0.01) (Fig 5). There was no difference in terms of hospital stay/hours (WMD: 1.13; 95% CI:-1.37 to 3.64; *p* = 0.38) and VAS (WMD: 0.25; 95% CI:-0.27 to 0.77; *p* = 0.34) (Fig 6) between stented and unstented groups. The unplanned readmission rate after discharge was significantly higher in the unstented group (OR: 0.54; 95% CI: 0.34 to 0.87; *p* = 0.01) (Fig 7).

### Outcomes of complications

**Fig 8:**
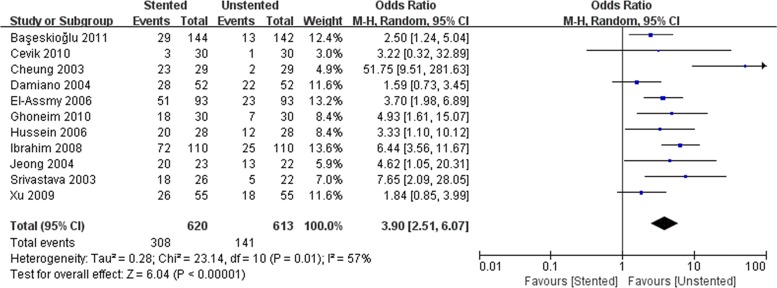
Forest plot and meta-analysis of dysuria rate.

**Fig 9:**
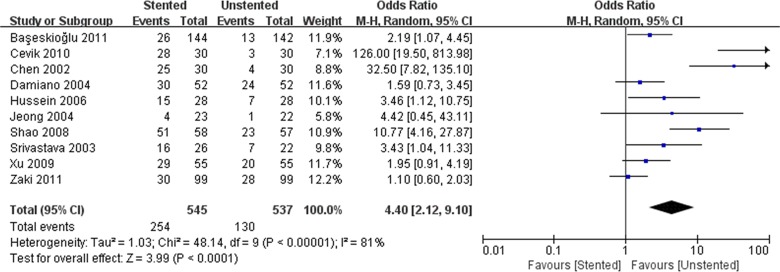
Forest plot and meta-analysis of irritative symptom rate.

**Fig 10:**
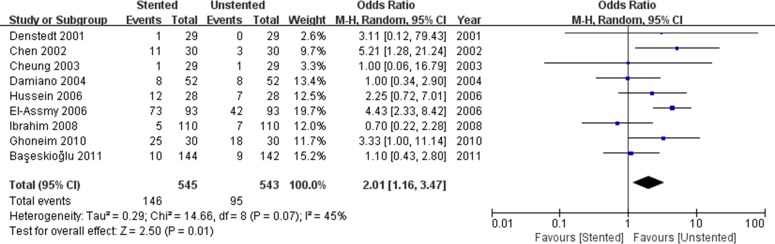
Forest plot and meta-analysis of urinary infection rate.

**Fig 11:**
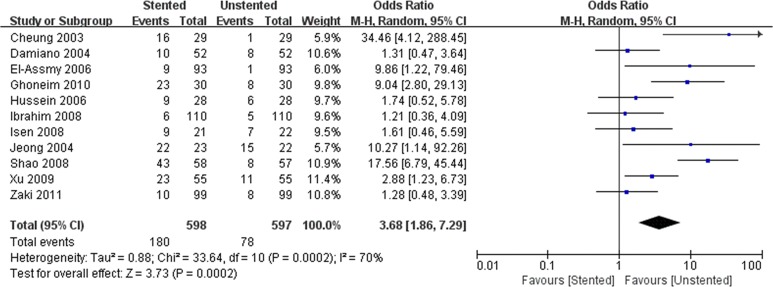
Forest plot and meta-analysis of hematuria rate.

**Fig 12:**
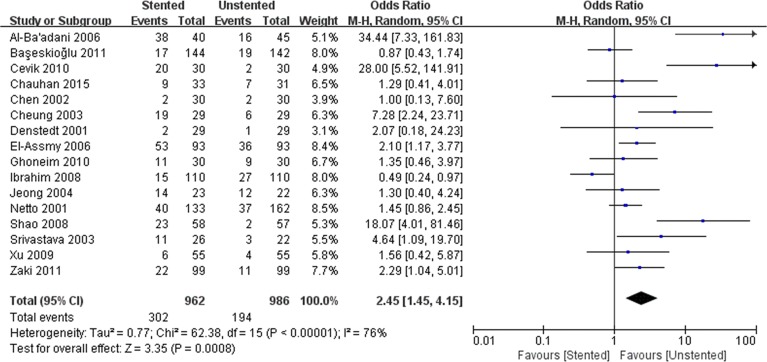
Forest plot and meta-analysis of pain rate.

We pooled data related to complications in the included studies. The most common complications were pain, dysuria, urinary infection, irritative symptom bladder symptoms, hematuria and fever. Long-term complications often manifested as ureteral strictures. The results showed a higher incidence of lower urinary tract symptom (LUTS) in the stented group including dysuria (OR: 3.90; 95% CI: 2.51 to 6.07; *p* < 0.001) (Fig 8) and irritation (OR: 4.40; 95% CI: 2.19 to 9.10; *p* < 0.001) (Fig 9). The incidence of urinary infection (OR: 2.01; 95% CI: 1.16 to 3.47; *p* = 0.01) (Fig 10) and hematuria (OR: 3.68; 95% CI: 1.86 to 7.29; *p* < 0.001) (Fig 11) was also higher in the stented group. Flank pain or voiding pain occurred more frequently in patients with stents (OR: 2.45; 95% CI: 1.45 to 4.15; *p* < 0.001) (Fig 12). No significant differences were found in fever rate (OR: 0.78; 95% CI: 0.52 to 1.18; *p* = 0.25) and ureteral stricture rate (OR: 0.52; 95% CI: 0.20 to 1.13; *p* = 0.17) between the two groups.

### Subgroup analysis

The results of subgroup analysis are displayed in Tables 3 and 4. According to the subgroup analysis based on publication years, only the results of readmission and infection rate varied from the original meta-analysis, without any significant differences between stenting and non-stenting groups. The stone free rate and infection rate were significantly different, after separating ESWL and URL-treated groups. Variables including operation time, hospital stay, VAS and other complications were not different from the original results, indicating that our results were not significantly influenced by lithotripsy and the treatment duration in the included studies.

**Table 3:** Subgroup analysis of trials in the last 10 years: stenting vs. non-stenting.

Outcome of interest	No. of studies	No. of patients	OR/WMD (95% CI)^†^	p-value	Study heterogeneity			
		Stented/Unstented			Chi^2^	df	I^2^	p-value
Operation time, min	11	689/686	4.18 [1.05, 7.31]^†^	**0.009**	99.45	10	90%	**<0.001**
Readmission rate	6	462/460	0.74 [0.42, 1.31]	0.30	5.42	5	8%	0.37
Hospital stay, day	4	119/125	1.97 [-0.12, 4.05]^†^	0.06	4.41	3	32%	0.22
Stone free rate	6	318/339	0.53 [0.31, 0.89]	**0.02**	2.14	5	0%	0.83
Visual analogue score	6	352/345	0.40 [-0.08, 0.88]^†^	0.10	38.15	5	87%	**<0.001**
Pain rate	10	692/692	2.67 [1.26, 5.65]	**0.01**	54.26	9	83%	**<0.001**
Dysuria rate	7	490/488	3.60 [2.66, 4.86]	**<0.001**	7.94	6	24%	0.24
Hematuria rate	8	494/494	3.31 [1.55, 7.05]	**0.002**	24.55	7	71%	**<0.001**
Irritative symptom rate	6	414/411	4.23 [1.64, 10.91]	**0.003**	34.13	5	85%	**<0.001**
Urinary infection rate	5	405/403	2.01 [0.98, 4.14]	0.06	10.66	4	62%	**0.03**
Fever rate	6	352/352	0.83 [0.51, 1.35]	0.45	0.77	5	0%	0.98

CI = confidence interval; OR = odds ratio; WMD = weighted mean difference

^†^Values of WMD.

Statistically significant results are shown in bold.

**Table 4:** Subgroup analysis of trials by URL and ESWL: stenting vs. non-stenting.

Outcome of interest	No. of studies	No. of patients	OR/WMD (95% CI)^†^	p-value	Study heterogeneity			
		stented/unstented			Chi^2^	df	I^2^	p-value
URL subgroup								
Operation time, min	16	958/980	4.93 [2.02, 7.84]^†^	**<0.001**	139.86	15	89%	**<0.001**
Readmission rate	9	596/595	0.54 [0.34, 0.87]	**0.01**	12.73	8	37%	0.12
Hospital stay, days	5	171/177	1.13 [-1.37, 3.64]^†^	0.38	8.98	4	55%	**0.06**
Stone-free rate	5	287/321	0.61 [0.22, 1.70]	0.35	2.80	4	0%	0.59
VAS	11	518/507	0.25 [-0.27, 0.77]^†^	0.34	102.55	10	90%	**<0.001**
Pain rate	14	839/863	2.69 [1.43, 5.06]	**0.002**	62.03	13	79%	**<0.001**
Dysuria rate	9	497/490	3.97 [2.24, 7.01]	**<0.001**	22.85	8	65%	**0.004**
Hematuria rate	9	475/474	3.09 [1.45, 6.60]	**0.003**	29.17	8	73%	**<0.001**
Irritative symptom rate	10	545/537	4.40 [2.12, 9.10]	**<0.001**	48.14	9	81%	**<0.001**
Urinary infection rate	7	422/420	1.44 [0.90, 2.30]	0.13	6.29	6	5%	0.39
Fever rate	7	403/403	0.79 [0.52, 1.21]	0.28	1.08	6	0%	0.98
ESWL subgroup								
Stone-free rate	3	193/209	0.53 [0.31, 0.92]	**0.02**	1.73	2	0%	0.42
Pain rate	2	123/123	1.90 [1.13, 3.17]	**0.01**	0.49	1	0%	0.48
Dysuria rate	2	123/123	3.96 [2.30, 6.82]	**<0.001**	0.19	1	0%	0.66
Hematuria rate	2	123/123	9.30 [3.28, 26.42]	**<0.001**	0.01	1	0%	0.94
Urinary infection rate	2	123/123	4.16 [2.36, 7.33]	**<0.001**	0.17	1	0%	0.68

CI = confidence interval; OR = odds ratio; WMD = weighted mean difference

†Values of WMD.

Statistically significant results are shown in bold.

### Sensitivity analysis and publication bias

Sensitivity analysis was performed by deleting one study each time and presented the results by Galbraith’s plots and L’Abbe plots (S1–S21 Figs). When the studies of Damiano [18] (OR:0.82, 95% CI: 0.51 to 1.30, *p* = 0.40) and Borboroglu [7] (OR:0.68, 95% CI: 0.43 to 1.06, *p* = 0.09) were omitted, the readmission rate was not significantly different between the two groups. Omission of the study of Sfoungaristos [31] yielded no significant difference in stone-free rate between the two groups (OR: 0.65, 95% CI: 0.35–1.21, *p* = 0.18). No other parameter in the pooled comparison between the two groups was significantly influenced by deleting any single study, indicating that the results of our meta-analysis were stable. Egger’s and Begg’s tests were used to assess the publication bias of included studies. Significant publication bias only existed in the comparison between irritative symptoms and pain (Table 5 and S22–S33 Figs).

**Table 5:** Results of Egger’s and Begg’s tests.

Outcome of interest	Number of included trials	P value of Egger’s test	P value of Begg’s test
Operation time	16	0.6561	0.3679
Readmission	9	0.3147	0.0953
Hospital stay	5	0.2651	0.3272
Stone-free rate	8	0.8271	0.6207
VAS	11	0.8563	0.8153
Dysuria	11	0.3362	0.4835
Urinary infection	9	0.48	0.6767
Dysuria	11	0.3362	0.4835
Hematuria	11	0.3426	0.4835
Pain	16	**0.0439**	**0.0244**
Irritative symptoms	10	**0.0101**	**0.0254**
Fever	8	0.5988	0.6207
Stricture	4	Not available	Not available

Statistically significant results are shown in bold. Egger’s and Begg’s tests are not available when fewer than 5 trials were included.

## Discussion

Advances in lithotripsy suggest that ESWL and URL are the first-line treatment for ureteral stones [3]. Routine ureteral stenting was the standard practice before and after ESWL and URL were developed, decades ago [34,35]. According to the current guidelines, a double J stent reduces the risk of renal colic and obstruction, but does not reduce steinstrasse formation or infective complications. Stents are recommended for patients who are at increased risk of complications such as ureteral trauma, residual fragments, bleeding, perforation, urinary tract infection, or pregnancy, and in all doubtful cases, to avoid stressful emergencies [3]. The previous meta-analyses in 2011 suggested that ureteral stents were not necessary after uncomplicated URL, because of LUTS and pain without improvement in stone-free rate or unplanned medical visits [36,37]. However, because of the lack of an established standard, the choice of stents for the treatment of ureteral stones is often a matter of surgical preference and experience. A survey carried out last year showed that 63% of the surgeons still routinely stented patients following URL [38]. Although ureteral stents are used differently in ESWL and URL, the function and purpose are similar. Therefore, it is reasonable to combine the trials of the two types of procedures. To our knowledge, our meta-analysis is the first of its kind that evaluates the benefits and disadvantages of ureteral stenting in ESWL and URL together. The size and duration of stents also significantly influences the clinical results, which varied in different trials. However, there is still no widely accepted standard of the diameter and duration of ureteral stents. Therefore, we merely focused on the indications for ureteral stenting.

Our meta-analysis showed acceptable baseline characteristics with no significant differences in age, gender, stone size or location between stented and unstented groups, suggesting the absence of any effect on perioperative or postoperative parameters. Stenting is of limited benefit in stone clearance and pain relief, and is associated with far greater complications including dysuria, urgency, hematuria and urinary tract infection (UTI). Compared with previous meta-analyses [36,37], postoperative VAS showed no significant difference between the two groups in our study. Nevertheless, stenting appeared to be safer with a lower incidence of re-hospitalization.

The results suggested that the stone-free rate might be influenced by the implantation of ureteral stents. It is well known that ureteroscopic surgery using holmium laser or pneumatic energy is a mature technology for the fragmentation and removal of ureteral stones, with nearly 100% stone-free rate irrespective of stenting [13,15]. ESWL is a safe and convenient approach without the need for anesthesia or hospitalization. However, more than one procedure is often needed to achieve complete stone clearance. In our study, the difference between the two groups in terms of stone-free rate was more obvious among patients undergoing ESWL. For example, in the study of El-Assmy the stone-free rate was 84.9% in the stented group compared with 91.4% in the unstented group [21]. In the study of Sfoungaristos, the rate was 68.6% versus 83.7%, respectively [31]. Therefore, the comparative significance was altered when the study of Sfoungaristos was deleted in the sensitive analysis. However, this result is questionable. In other studies, after ESWL, the stone-free rate was not significantly different between stented and unstented groups [6,29,39].

The stent-related complications are the most significant drawbacks of ureteral stenting in most patients [40]. As a foreign substance, ureteral stents trigger postoperative flank pain. Patients need anesthesia to relieve the discomfort, which is explained by reflux and higher intra-pelvic pressure especially during voiding [41]. Interestingly, based on the study results, there was no significant difference between stented and unstented groups in the mean visual analogue scores as approximately, only a third of all the patients in the stented group complained of pain, despite more painful attacks. Moreover, the pain decreased eventually in both stented and unstented groups [18,22]. In addition to pain, indwelling stents stimulate and irritate the bladder mucous membrane, resulting in a wide range of urinary symptoms including dysuria, urgency, hematuria and UTI. Experiments showed that long-indwelling stents cause ureteral wall edema, epithelial hyperplasia, destruction, and inflammatory cell reactions, suggesting their use only over the short term [42]. Usually 4 weeks after stent implantation, late complications appear such as hydronephrosis, stent migration, encrustation, fragmentation and breakage [43]. In addition, with longer indwelling time, there is a higher incidence of incrustation, infections, secondary stone formation and obstruction of the stented tract. The ideal duration of stenting is still unknown, and 1 to 4 weeks are usually recommended after lithotripsy [3]. In Djaladat’s study, stents with the end attached to a Foley’s catheter, and inserted in the urethra were removed only 24 h after operation, which effectively reduced early postoperative morbidities and also decreased pain and colic after discharge [44]. Our results demonstrated that ureteral strictures were not directly related to non-stenting. Removal of stents within a short term is considered safe.

In spite of all these stent-associated discomfort and risks, ureteral stents are safe after URL or ESWL. In our study, the rate of unplanned re-hospitalization was significantly higher in the unstented group, which was reported in 9 trials [7,16,18,23–25,28,30,33]. The result is questionable due to the varying outcome in the subgroup analysis based on the studies conducted in the last 10 years. However, it did not imply that the risk of uncontrolled complications was decreased. Patients needed readmission mostly because of severe flank pain, high fever or gross hematuria after discharge. Although the rate of postoperative pain and fever did not diminish in the stented group, the risk of severe complications was stably reduced by ureteral stenting. In Chandhoke’s study, the use of stents also resulted in fewer hospital readmissions and emergency room visits after the treatment for both renal and ureter stones [6]. Moreover, insertion of double J stents for 4 weeks after URL significantly decreased the frequency of ureteral colic [4]. In addition, the length of hospital stay was similar in the two groups, suggesting that stenting did not affect postoperative recovery. Overall, the re-hospitalization rate should be weighed against the morbidity associated with stents, especially in patients with higher risks. Most stent-related complications are easily and effectively resolved following stent removal [43]. It is still too early to conclude that stenting was unnecessary for the treatment of ureteral stones. The cost of saving and increased comfort should be weighed against the potential for severe post-discharge complications. Alpha-blockers effectively reduced the morbidity of ureteral stents [45,46]. A meta-analysis showed that, tamsulosin and alfuzosin, which were the most commonly applied drugs, had the similar function to relief the stents-related discomfort [47].

We should admit several limitations associated with our study. First, only those studies published in English were pooled in our meta-analysis, and a few related studies published in other languages were missed. Second, although the funnel plots only showed publication bias in the comparison of hematuria and dysuria, the role of bias in our study could not be completely excluded. Third, the length of follow-up, the power of lithotripsy and the diameters and duration of stent implantation were not similar across the different trials. The influence of heterogeneity could not be evaluated. Fourth, the poor qualities of original trials led to low levels of heterogeneity in this study. Last, a study involving renal stones was excluded [6], and data could not be fully extracted due to the study design [4]. As a result, valuable data were inevitably missed.

## Conclusions

There is no consensus on the indications for ureteral stenting during the treatment of ureteral stones. Our meta-analysis based on 22 RCTs suggested that stenting was associated with more discomfort and LUTS including dysuria, hematuria, irritation and UTI. However, it is recommended in selected patients with relatively higher risk of unremitting pain and uncontrolled fever after discharge. Additional multi-center RCTs with large sample size and high quality are needed, including detailed data involving patients’ clinical demographics, standard surgical procedures and follow-up at periodic intervals after lithotripsy.

## Supporting Information

S1 Checklist PRISMA 2009 Checklist. (DOC)

S1 Fig Random Galbraith’s plot of operation time. (PDF)

S2 Fig Fixed Galbraith’s plot of readmission rate. (PDF)

S3 Fig Fixed Galbraith’s plot of hospital stay. (PDF)

S4 Fig Random Galbraith’s plot of flank pain rate. (PDF)

S5 Fig Random Galbraith’s plot of VAS. (PDF)

S6 Fig Fixed Galbraith’s plot of stone free rate. (PDF)

S7 Fig Random Galbraith’s plot of dysuria rate. (PDF)

S8 Fig Random Galbraith’s plot of irritative symptom rate. (PDF)

S9 Fig Random Galbraith’s plot of infection rate. (PDF)

S10 Fig Fixed Galbraith’s plot of stricture rate. (PDF)

S11 Fig Random Galbraith’s plot of hematuria rate. (PDF)

S12 Fig 24: Fixed Galbraith’s plot of fever rate. (PDF)

S13 Fig L’Abbe plot of readmission rate. (PDF)

S14 Fig L’Abbe plot of pain rate. (PDF)

S15 Fig L’Abbe plot of stone free rate. (PDF)

S16 Fig L’Abbe plot of dysuria rate. (PDF)

S17 Fig L’Abbe plot of irritative symptom rate. (PDF)

S18 Fig L’Abbe plot of infection rate. (PDF)

S19 Fig L’Abbe plot of stricture rate. (PDF)

S20 Fig L’Abbe plot of hematuria rate. (PDF)

S21 Fig L’Abbe plot of fever rate. (PDF)

S22 Fig Funnel plot of operation time. (PDF)

S23 Fig Funnel plot of readmission rate. (PDF)

S24 Fig Funnel plot of hospital stay. (PDF)

S25 Fig Funnel plot of pain rate. (PDF)

S26 Fig Funnel plot of visual analogue score (VAS). (PDF)

S27 Fig Funnel plot of stone free rate. (PDF)

S28 Fig Funnel plot of dysuria rate. (PDF)

S29 Fig Funnel plot of irritative symptom rate. (PDF)

S30 Fig Funnel plot of urinary infection rate. (PDF)

S31 Fig Funnel plot of stricture rate. (PDF)

S32 Fig Funnel plot of hematuria rate. (PDF)

S33 Fig Funnel plot of fever rate. (PDF)

## References

[1] Lopez M, Hoppe B. History, epidemiology and regional diversities of urolithiasis. Pediatr Nephrol. 2010; 25: 49–59. 10.1007/s00467-008-0960-5 21476230 PMC2778769

[2] Stamatelou KK, Francis ME, Jones CA, Nyberg LM, Curhan GC. Time trends in reported prevalence of kidney stones in the United States: 1976–1994. Kidney Int. 2003; 63: 1817–1823. 10.1046/j.1523-1755.2003.00917.x 12675858

[3] Turk C, Petrik A, Sarica K, Seitz C, Skolarikos A, Straub M, et al EAU Guidelines on Interventional Treatment for Urolithiasis. Eur Urol. 2016; 69: 475–482. 10.1016/j.eururo.2015.07.041 26344917

[4] Aghamir SM, Mohammadi A, Farahmand H, Meysamie AP. Effects of prophylactic insertion of Double-J stents to decrease episodes of renal colic in patients with recurrent ureteral stones. J Endourol. 2008; 22: 435–437. 10.1089/end.2007.0163 18355138

[5] Chew BH, Seitz C. Impact of ureteral stenting in ureteroscopy. Curr Opin Urol. 2016; 26: 76–80. 10.1097/MOU.0000000000000234 26626886

[6] Chandhoke PS, Barqawi AZ, Wernecke C, RA. C-A. A randomized outcomes trial of ureteral stents for extracorporeal shock wave lithotripsy of solitary kidney or proximal ureteral stones. J Urol. 2002; 167: 1981–1983. 11956422

[7] Borboroglu PG, Amling CL, Schenkman NS, Monga M, Ward JF, Piper NY, et al Ureteral stenting after ureteroscopy for distal ureteral calculi: a multi-institutional prospective randomized controlled study assessing pain, outcomes and complications. J Urol. 2001; 166: 1651–1657. 11586195 10.1016/s0022-5347(05)65646-7

[8] Assimos D, Krambeck A, Miller NL, Monga M, Murad MH, Nelson CP, et al Surgical Management of Stones: American Urological Association/Endourological Society Guideline, PART I.J Urol. 2016;196(4):1153–60. 10.1016/j.juro.2016.05.090 27238616

[9] Moher D, Liberati A, Tetzlaff J, Altman DG, Group P (2009) Preferred reporting items for systematic reviews and meta-analyses: the PRISMA statement. PLoS Med 6: e1000097 10.1371/journal.pmed.1000097 19621072 PMC2707599

[10] Higgins J, Green S. Cochrane Handbook for Systematic Reviews of Interventions. Version 5.0.2. The Cochrane Collaboration 2009.

[11] Clarke M R. H. Bringing it all together: Lancet-Cochrane collaborate on systematic reviews. Lancet. 2001; 357: 1728. 10.1016/S0140-6736(00)04934-5 11403806

[12] Hozo SP, Djulbegovic B, Hozo I. Estimating the mean and variance from the median, range, and the size of a sample. BMC Med Res Methodol. 2005; 5: 13 10.1186/1471-2288-5-13 15840177 PMC1097734

[13] Denstedt JD, Wollin TA, Sofer M, Nott L, Weir M, RJ. DAH. A prospective randomized controlled trial comparing nonstented versus stented ureteroscopic lithotripsy. J Urol. 2001; 165: 1419–1422. 11342889

[14] Netto NR Jr, Ikonomidis J, C. Z. Routine ureteral stenting after ureteroscopy for ureteral lithiasis: is it really necessary? J Urol. 2001; 166: 1252–1254. 11547052

[15] Chen YT, Chen J, Wong WY, Yang SS, Hsieh CH, CC. W. Is ureteral stenting necessary after uncomplicated ureteroscopic lithotripsy? A prospective, randomized controlled trial. J Urol 2002; 167: 1977–1980. 11956421

[16] Cheung MC, Lee F, Leung YL, Wong BB, Tam PC. A prospective randomized controlled trial on ureteral stenting after ureteroscopic holmium laser lithotripsy. J Urol. 2003; 169: 1257–1260. 10.1097/01.ju.0000053763.30693.ef 12629338

[17] Srivastava A, Gupta R, Kumar A, Kapoor R, A. M. Routine stenting after ureteroscopy for distal ureteral calculi is unnecessary: results of a randomized controlled trial. J Endourol 2003; 17: 871–874. 10.1089/089277903772036172 14744352

[18] Damiano R, Autorino R, Esposito C, Cantiello F, Sacco R, de Sio M, et al Stent positioning after ureteroscopy for urinary calculi: the question is still open. Eur Urol. 2004; 46: 381–387; discussion 387–388. 10.1016/j.eururo.2004.04.004 15306112

[19] Jeong H, Kwak C, SE. L. Ureteric stenting after ureteroscopy for ureteric stones: a prospective randomized study assessing symptoms and complications BJU Int. 2004; 93: 1032–1034. 10.1111/j.1464-410X.2004.4776a.x 15142158

[20] Al-Ba'adani T, Ghilan A, El-Nono I, Alwan M, A. B. Whether post-ureteroscopy stenting is necessary or not? Saudi Med J. 2006; 27: 845–848. 16758048

[21] El-Assmy A, El-Nahas AR, Sheir KZ. Is pre-shock wave lithotripsy stenting necessary for ureteral stones with moderate or severe hydronephrosis? J Urol. 2006; 176: 2059–2062; discussion 2062. 10.1016/j.juro.2006.07.022 17070256

[22] Hussein A, Rifaat E, Zaki A, M. A-N. Stenting versus non-stenting after non-complicated ureteroscopic manipulation of stones in bilharzial ureters. Int J Urol 2006; 13: 886–890. 10.1111/j.1442-2042.2006.01434.x 16882049

[23] Ibrahim HM, Al-Kandari AM, Shaaban HS, Elshebini YH, Shokeir AA. Role of ureteral stenting after uncomplicated ureteroscopy for distal ureteral stones: a randomized, controlled trial. J Urol. 2008; 180: 961–965. 10.1016/j.juro.2008.05.030 18639269

[24] Isen K, Bogatekin S, Em S, Ergin H, Kilic V. Is routine ureteral stenting necessary after uncomplicated ureteroscopic lithotripsy for lower ureteral stones larger than 1 cm? Urol Res. 2008; 36: 115–119. 10.1007/s00240-008-0135-7 18385992

[25] Shao Y, Zhuo J, Sun XW, Wen W, Liu HT, Xia SJ. Nonstented versus routine stented ureteroscopic holmium laser lithotripsy: a prospective randomized trial. Urol Res. 2008; 36: 259–263. 10.1007/s00240-008-0153-5 18797859

[26] Wang CJ, Huang SW, Chang CH. Indications of stented uncomplicated ureteroscopic lithotripsy: a prospective randomized controlled study. Urol Res. 2009; 37: 83–88. 10.1007/s00240-009-0175-7 19183976

[27] Xu Y, Wei Q, LR. L. A prospective randomized trial comparing non-stented versus routine stented ureteroscopic holmium laser lithotripsy. Saudi Med J. 2009; 30: 1276–1280. 19838433

[28] Cevik I, Dillioglugil O, Akdas A, Y. S. Is stent placement necessary after uncomplicated ureteroscopy for removal of impacted ureteral stones? J Endourol 2010; 24: 1263–1267. 10.1089/end.2009.0153 20615145

[29] Ghoneim IA, El-Ghoneimy MN, El-Naggar AE, Hammoud KM, El-Gammal MY, Morsi AA. Extracorporeal shock wave lithotripsy in impacted upper ureteral stones: a prospective randomized comparison between stented and non-stented techniques. Urology. 2010; 75: 45–50. 10.1016/j.urology.2009.06.071 19811806

[30] Baseskioglu B, Sofikerim M, Demirtas A, Yenilmez A, Kaya C, Can C. Is ureteral stenting really necessary after ureteroscopic lithotripsy with balloon dilatation of ureteral orifice? A multi-institutional randomized controlled study. World J Urol. 2011; 29: 731–736. 10.1007/s00345-011-0697-9 21590466

[31] Sfoungaristos S, Polimeros N, Kavouras A, Perimenis P. Stenting or not prior to extracorporeal shockwave lithotripsy for ureteral stones? Results of a prospective randomized study. Int Urol Nephrol. 2012; 44: 731–737. 10.1007/s11255-011-0062-3 21960371

[32] Chauhan VS, Bansal R, Ahuja M. Comparison of efficacy and tolerance of short-duration open-ended ureteral catheter drainage and tamsulosin administration to indwelling double J stents following ureteroscopic removal of stones. Hong Kong Med J. 2015; 21: 124–130. 10.12809/hkmj144292 25756274

[33] Zaki M.R, Salman A, Chaudhary A.H, Asif K, M.U. R. Is DJ stenting still needed after uncomplicated ureteroscopic lithotripsy? A randomized controlled trial. Pak J Med Sci. 2011; 5: 121–124.

[34] Abdel-Khalek M, Sheir K, Elsobky E, Showkey S, Kenawy M. Prognostic factors for extracorporeal shock-wave lithotripsy of ureteric stones—a multivariate analysis study. Scand J Urol Nephrol. 2003; 37: 413–418. 10.1080/00365590310006255 14594691

[35] Harmon WJ, Sershon PD, Blute ML, Patterson DE, JW. S. Ureteroscopy: current practice and long-term complications. J Urol. 1997; 157: 28–32. 8976208 10.1016/s0022-5347(01)65272-8

[36] Pengfei S, Yutao L, Jie Y, Wuran W, Yi D, Hao Z, et al The results of ureteral stenting after ureteroscopic lithotripsy for ureteral calculi: a systematic review and meta-analysis. J Urol. 2011; 186: 1904–1909. 10.1016/j.juro.2011.06.066 21944085

[37] Song T, Liao B, Zheng S, Wei Q. Meta-analysis of postoperatively stenting or not in patients underwent ureteroscopic lithotripsy. Urol Res. 2012; 40: 67–77. 10.1007/s00240-011-0385-7 21573923

[38] Dauw CA, Simeon L, Alruwaily AF, Sanguedolce F, Hollingsworth JM, Roberts WW, et al Contemporary Practice Patterns of Flexible Ureteroscopy for Treating Renal Stones: Results of a Worldwide Survey. J Endourol. 2015; 29: 1221–1230. 10.1089/end.2015.0260 26154856

[39] Wang M, Shi Q, Wang X, Yang K, Yang R. Prediction of outcome of extracorporeal shock wave lithotripsy in the management of ureteric calculi. Urol Res. 2011; 39: 51–57. 10.1007/s00240-010-0274-5 20401653

[40] Keeley FX Jr., Timoney AG. Routine stenting after ureteroscopy: think again. Eur Urol. 2007; 52: 642–644. 10.1016/j.eururo.2007.01.087 17275989

[41] Joshi HB, Stainthorpe A, MacDonagh RP, Keeley FX Jr, Timoney AG, MJ. B. Indwelling ureteral stents: evaluation of symptoms, quality of life and utility. J Urol. 2003; 169: 1065–1069. 10.1097/01.ju.0000048980.33855.90 12576847

[42] Lumiaho J, Heino A, Pietiläinen T, Ala-Opas M, Talja M, Välimaa T, et al The morphological, in situ effects of a self-reinforced bioabsorbable polylactide (SR-PLA 96) ureteric stent; an experimental study. J Urol 2000; 164: 1360–1363. 10992415

[43] Damiano R, Oliva A, Esposito C, De Sio M, Autorino R, M. DA. Early and late complications of double pigtail ureteral stent. Urol Int. 2002; 69: 136–140. 12187045 10.1159/000065563

[44] Djaladat H, Tajik P, Payandemehr P, Alehashemi S. Ureteral catheterization in uncomplicated ureterolithotripsy: a randomized, controlled trial. Eur Urol. 2007; 52: 836–841. 10.1016/j.eururo.2007.01.042 17258387

[45] Singh I, Tripathy S, Agrawal V. Efficacy of tamsulosin hydrochloride in relieving "double-J ureteral stent-related morbidity": a randomized placebo controlled clinical study. Int Urol Nephrol. 2014; 46: 2279–2283. 10.1007/s11255-014-0825-8 25201459

[46] Lamb AD, Vowler SL, Johnston R, Dunn N, Wiseman OJ. Meta-analysis showing the beneficial effect of alpha-blockers on ureteric stent discomfort. BJU Int. 2011; 108: 1894–1902. 10.1111/j.1464-410X.2011.10170.x 21453351

[47] Kwon JK, Cho KS, Oh CK, Kang DH, Lee H, Ham WS, et al The beneficial effect of alpha-blockers for ureteral stent-related discomfort: systematic review and network meta-analysis for alfuzosin versus tamsulosin versus placebo. BMC Uro. 2015;15:55 10.1186/s12894-015-0050-5 PMC4477492 26104313

