# Supplementary figures and images for: Meta-Analysis of Stenting versus Non-Stenting for the Treatment of Ureteral Stones
Source: PLoS One. 2017 Jan 9;12(1):e0167670. doi: 10.1371/journal.pone.0167670 (PMC5221881; doi:10.1371/journal.pone.0167670)

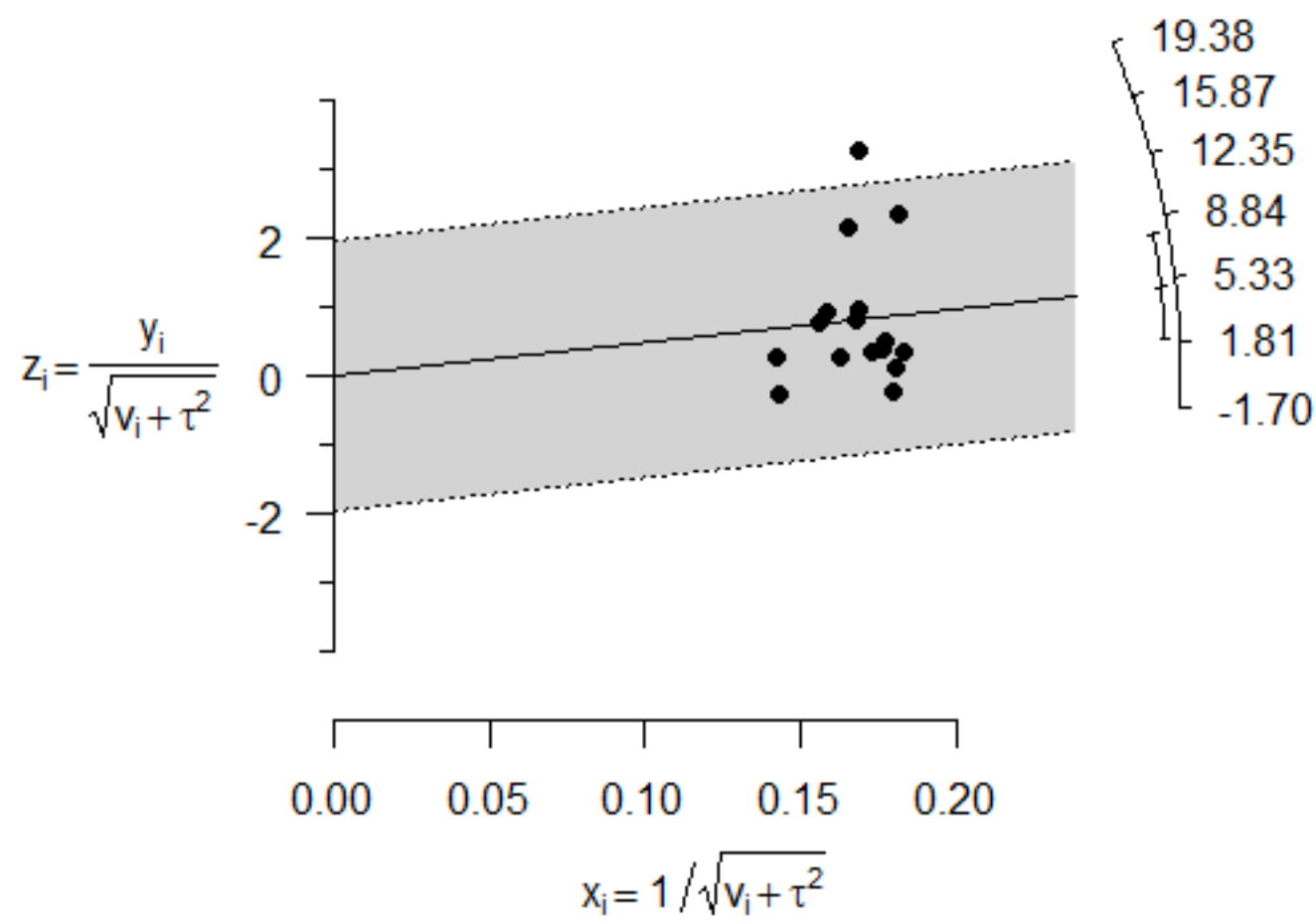

Supplement: S1 Fig — (PDF) [file pone.0167670.s002.pdf]

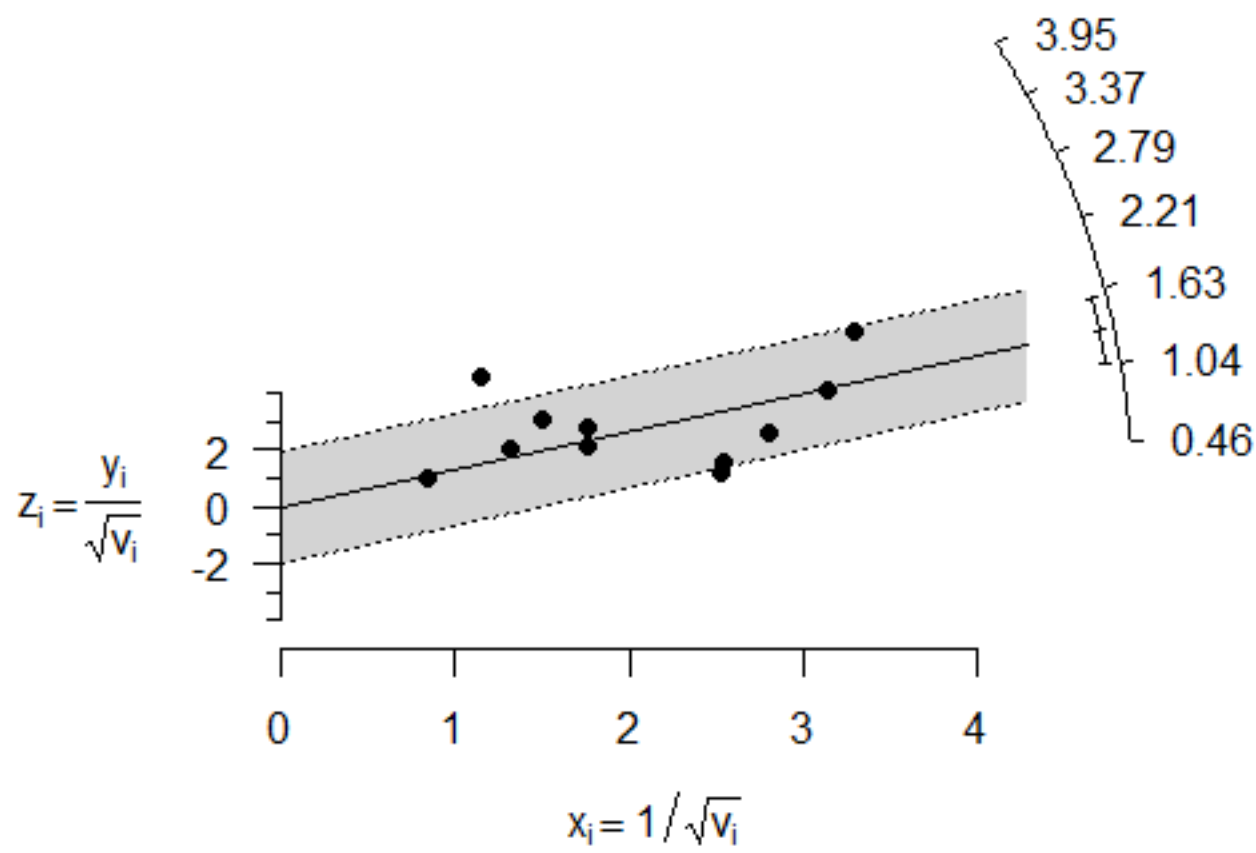

Supplement: S2 Fig — (PDF) [file pone.0167670.s003.pdf]

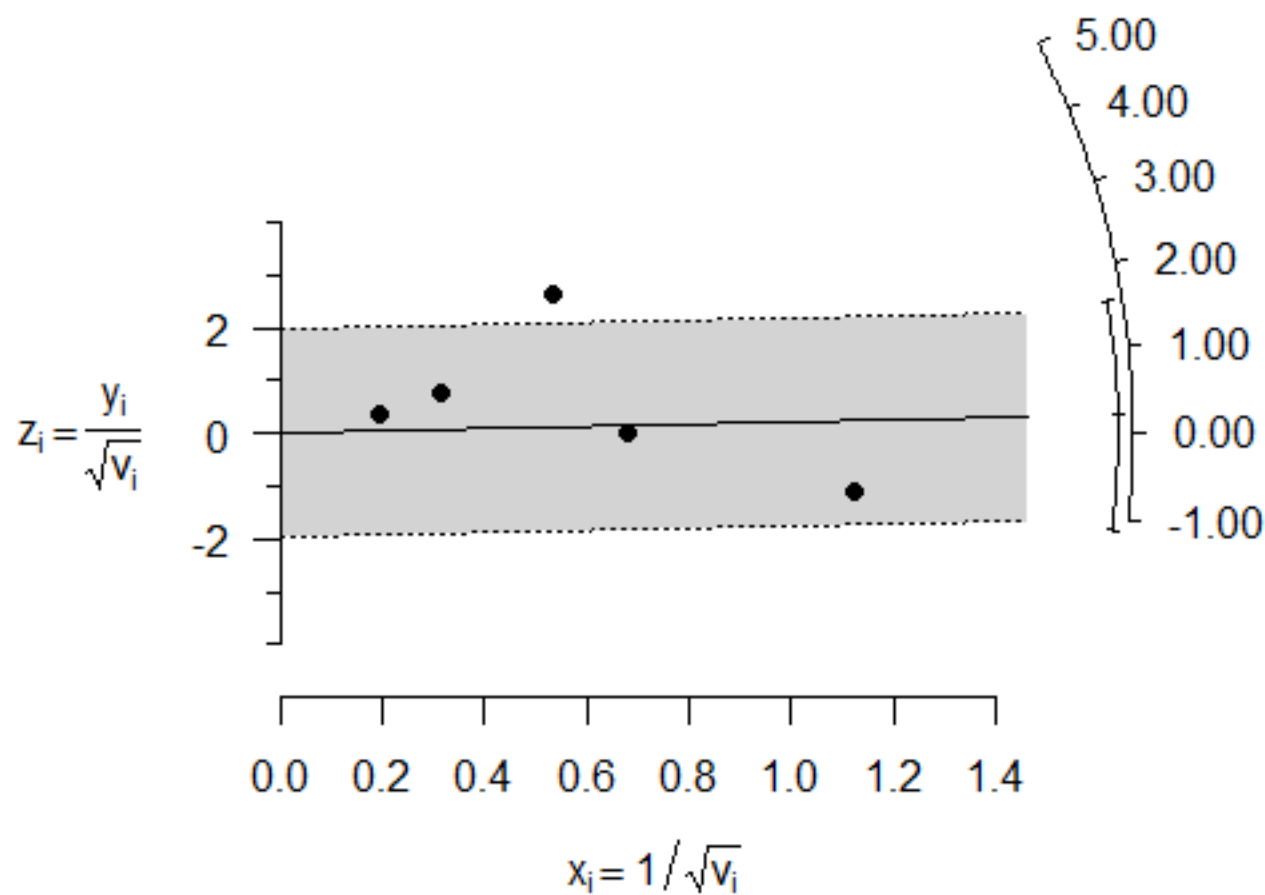

Supplement: S3 Fig — (PDF) [file pone.0167670.s004.pdf]

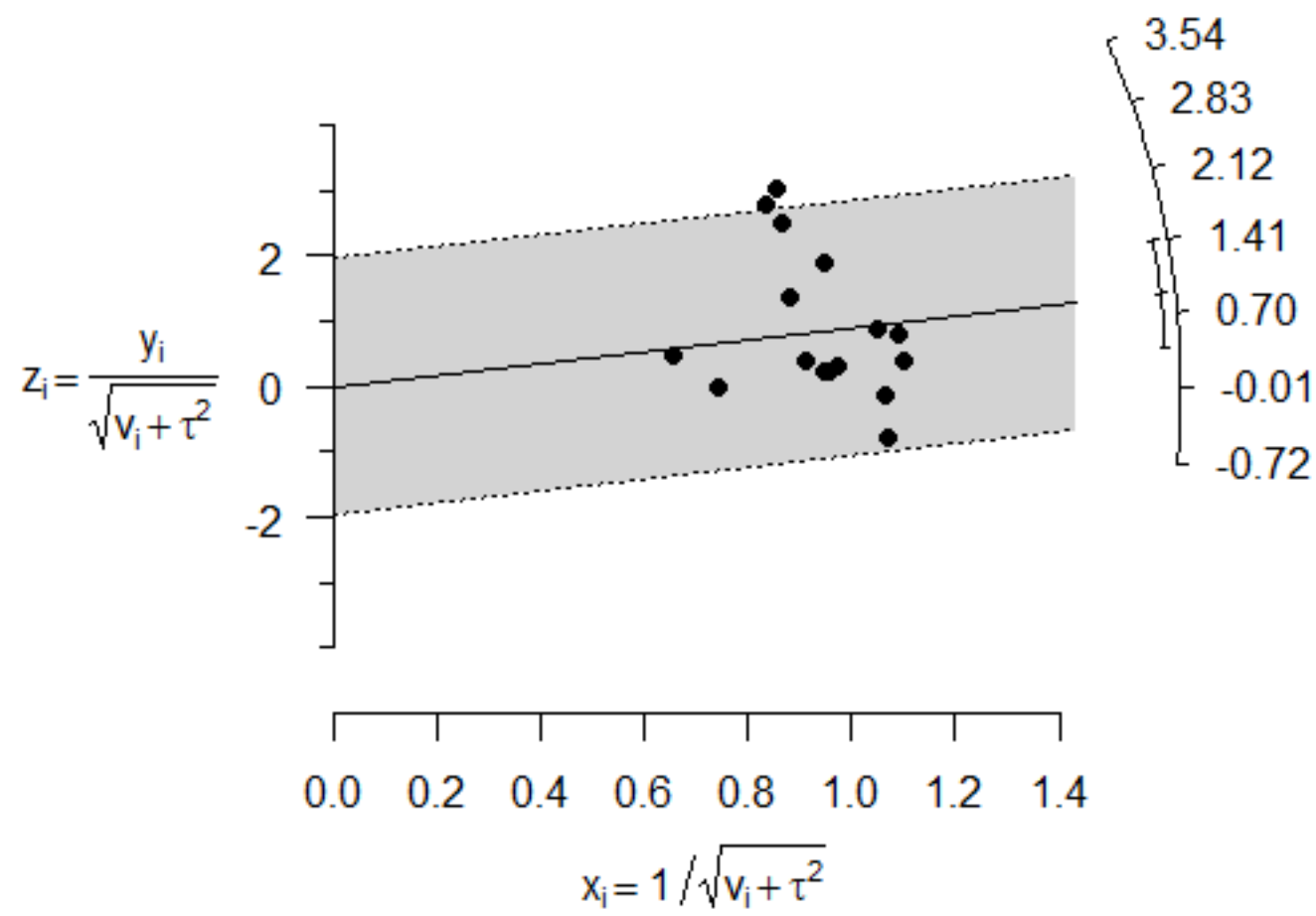

Supplement: S4 Fig — (PDF) [file pone.0167670.s005.pdf]

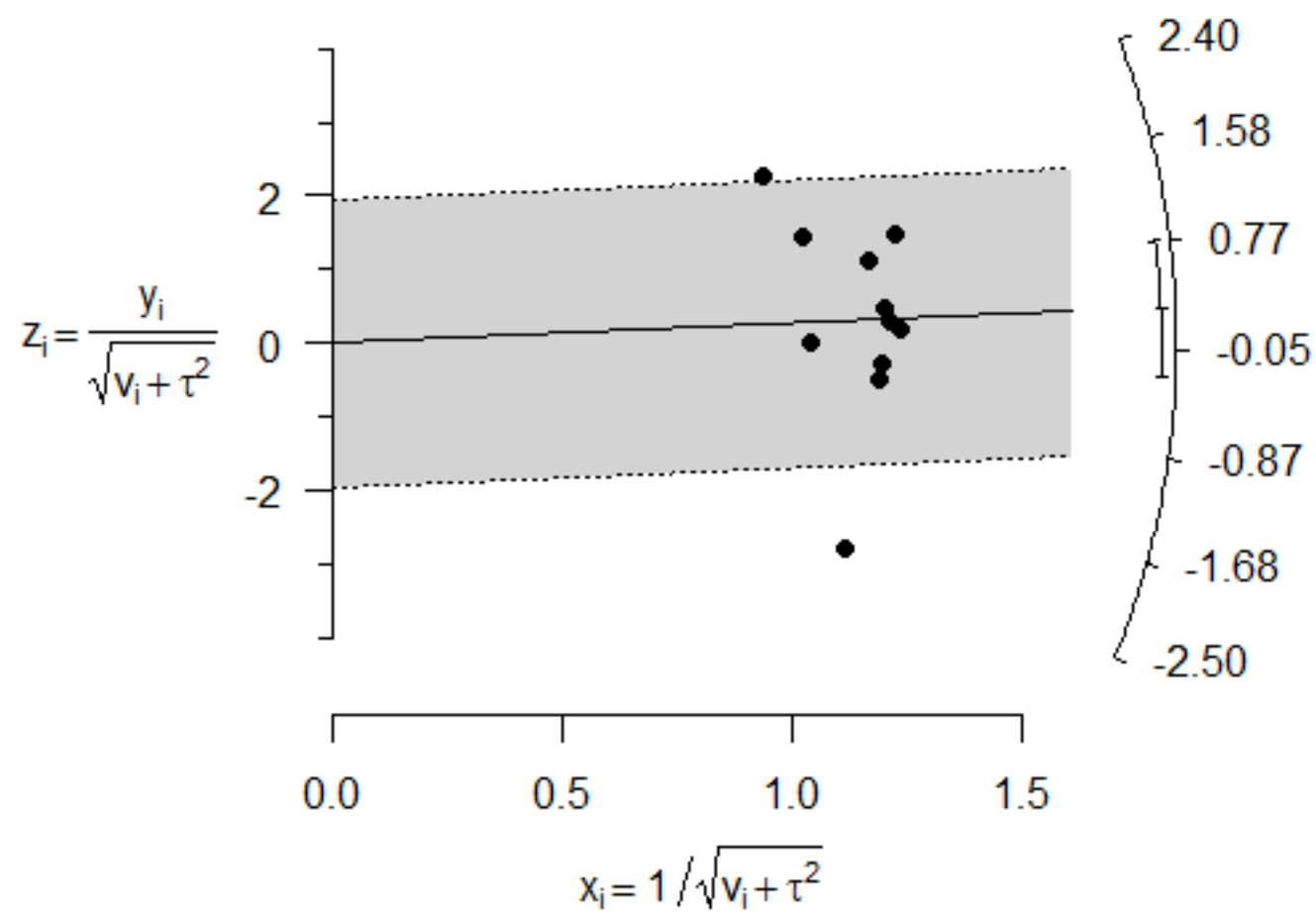

Supplement: S5 Fig — (PDF) [file pone.0167670.s006.pdf]

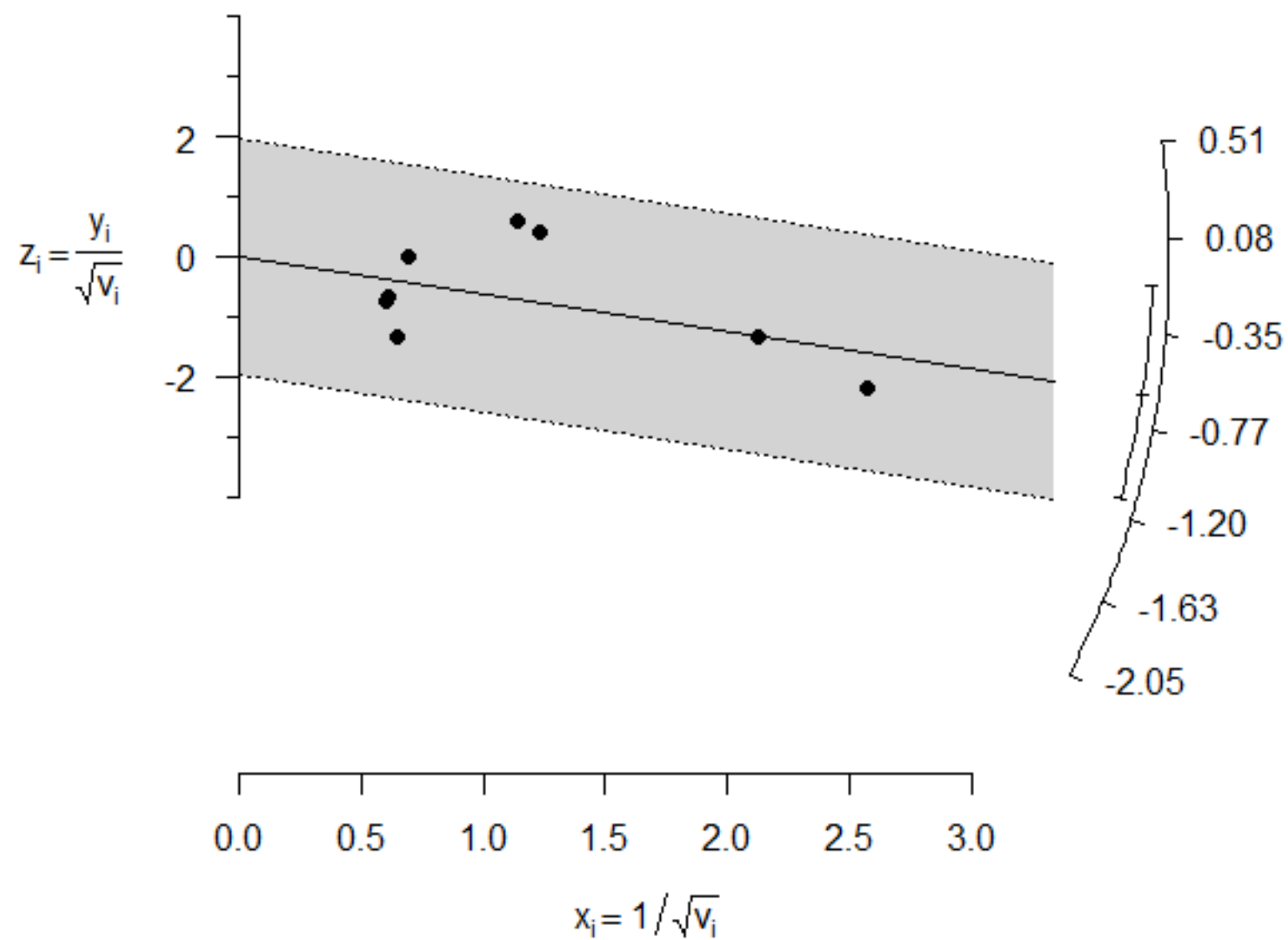

Supplement: S6 Fig — (PDF) [file pone.0167670.s007.pdf]

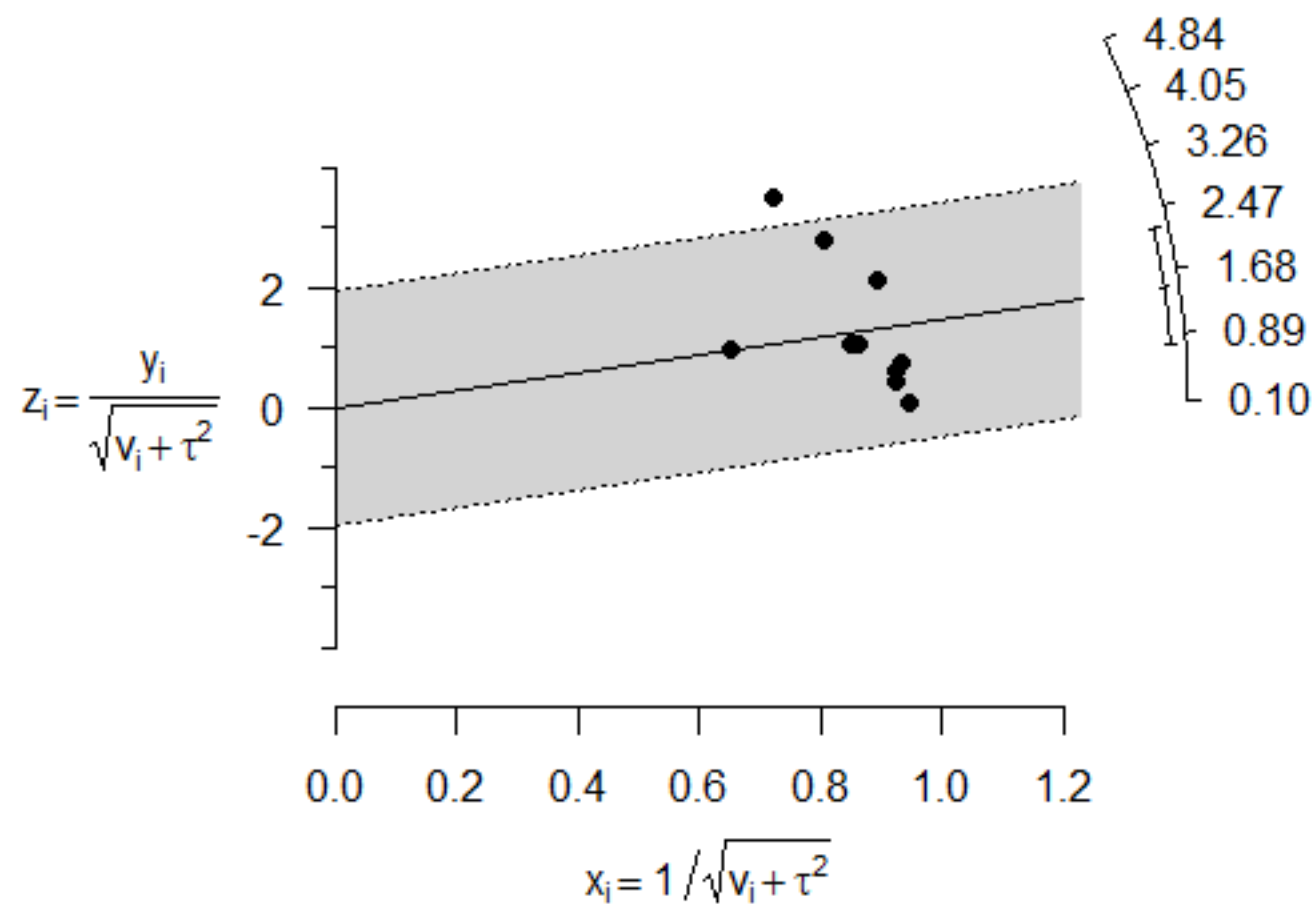

Supplement: S7 Fig — (PDF) [file pone.0167670.s008.pdf]

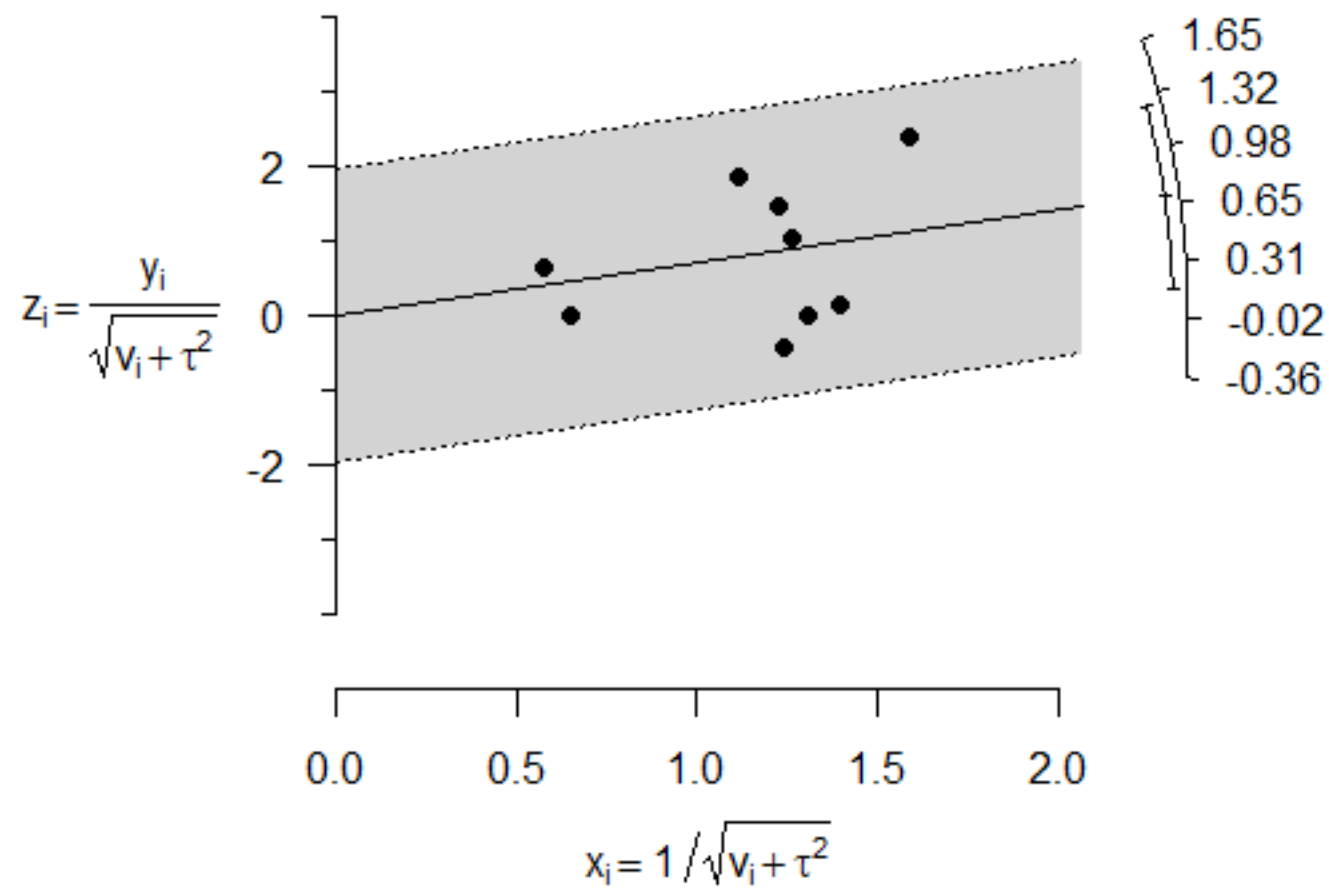

Supplement: S8 Fig — (PDF) [file pone.0167670.s009.pdf]

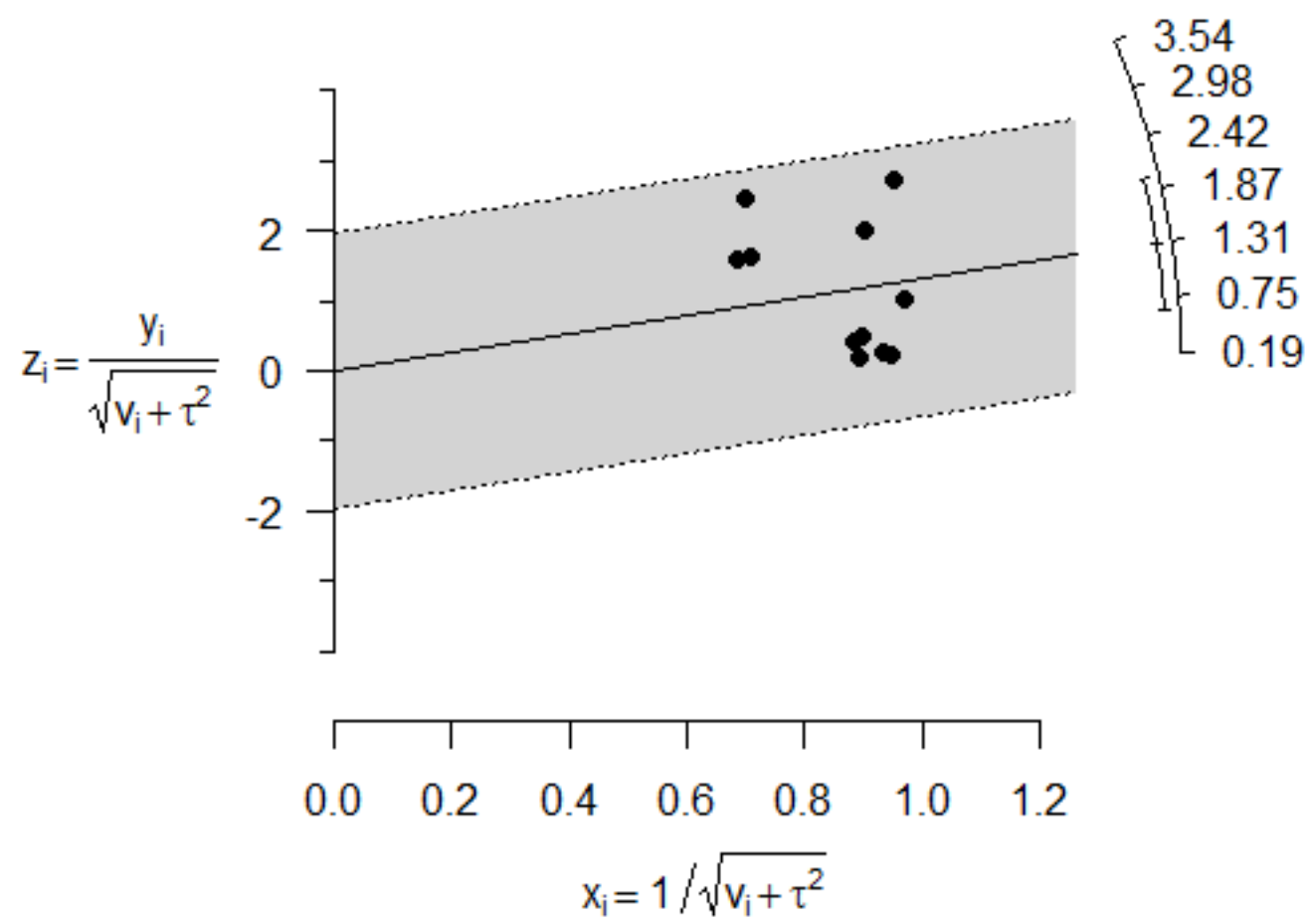

Supplement: S9 Fig — (PDF) [file pone.0167670.s010.pdf]

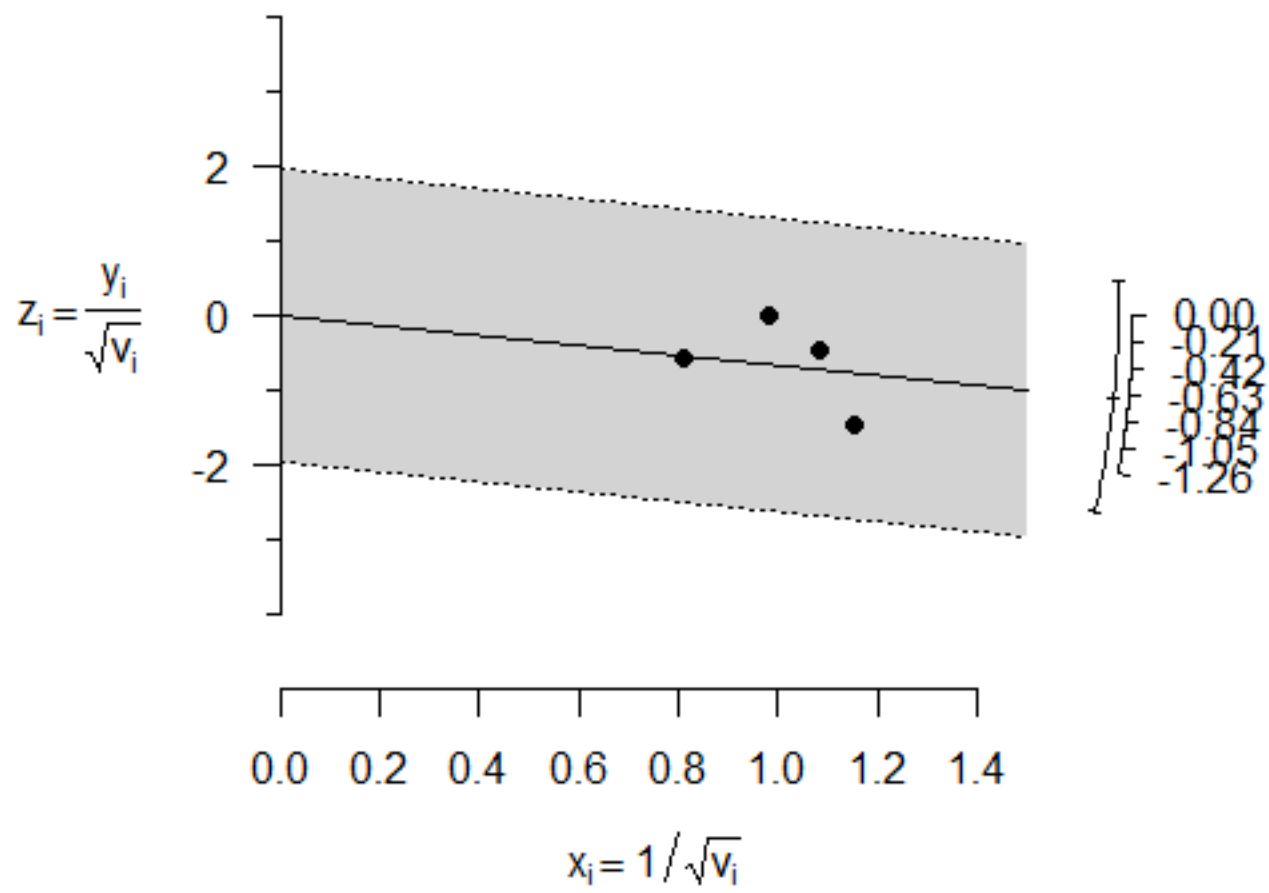

Supplement: S10 Fig — (PDF) [file pone.0167670.s011.pdf]

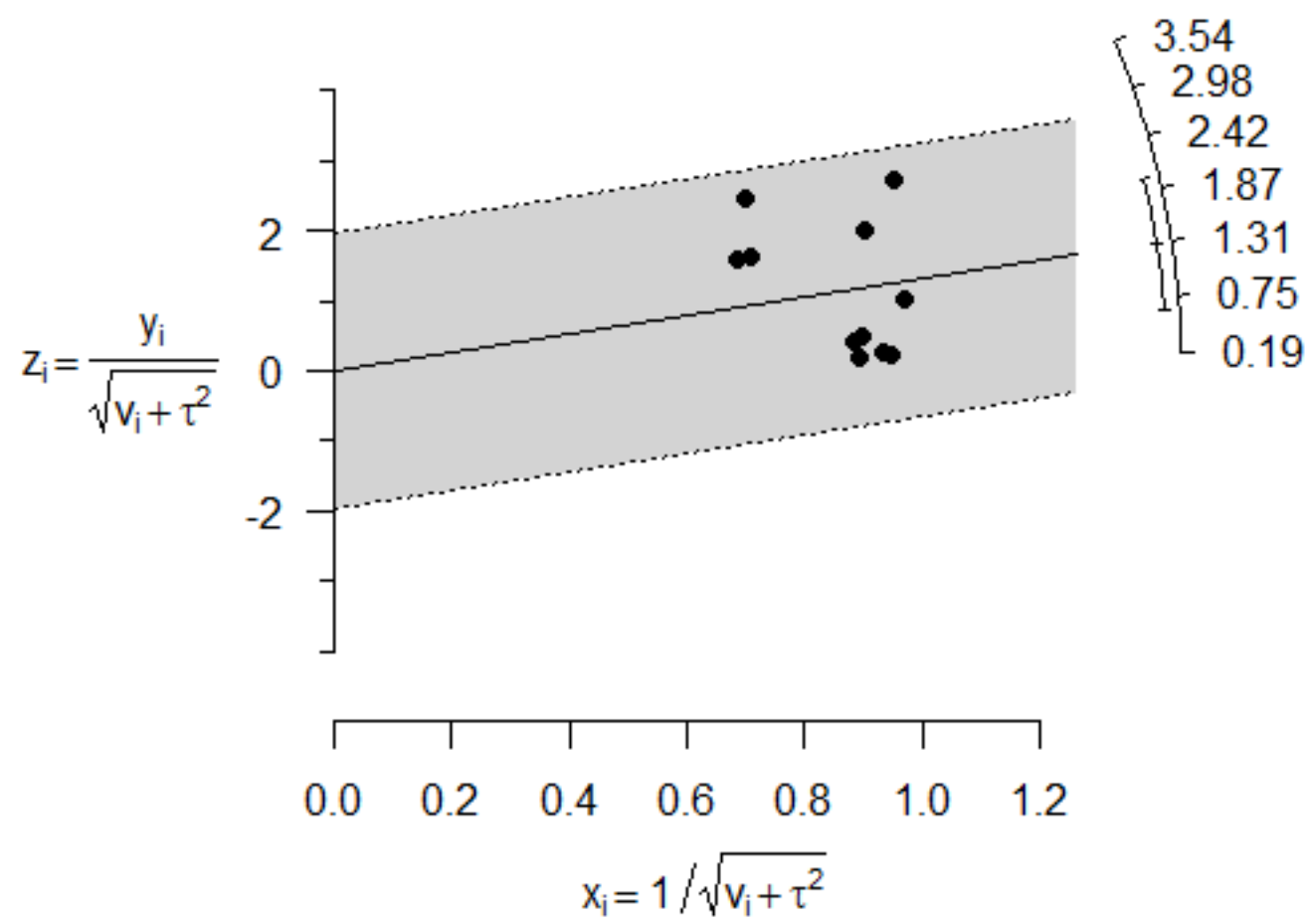

Supplement: S11 Fig — (PDF) [file pone.0167670.s012.pdf]

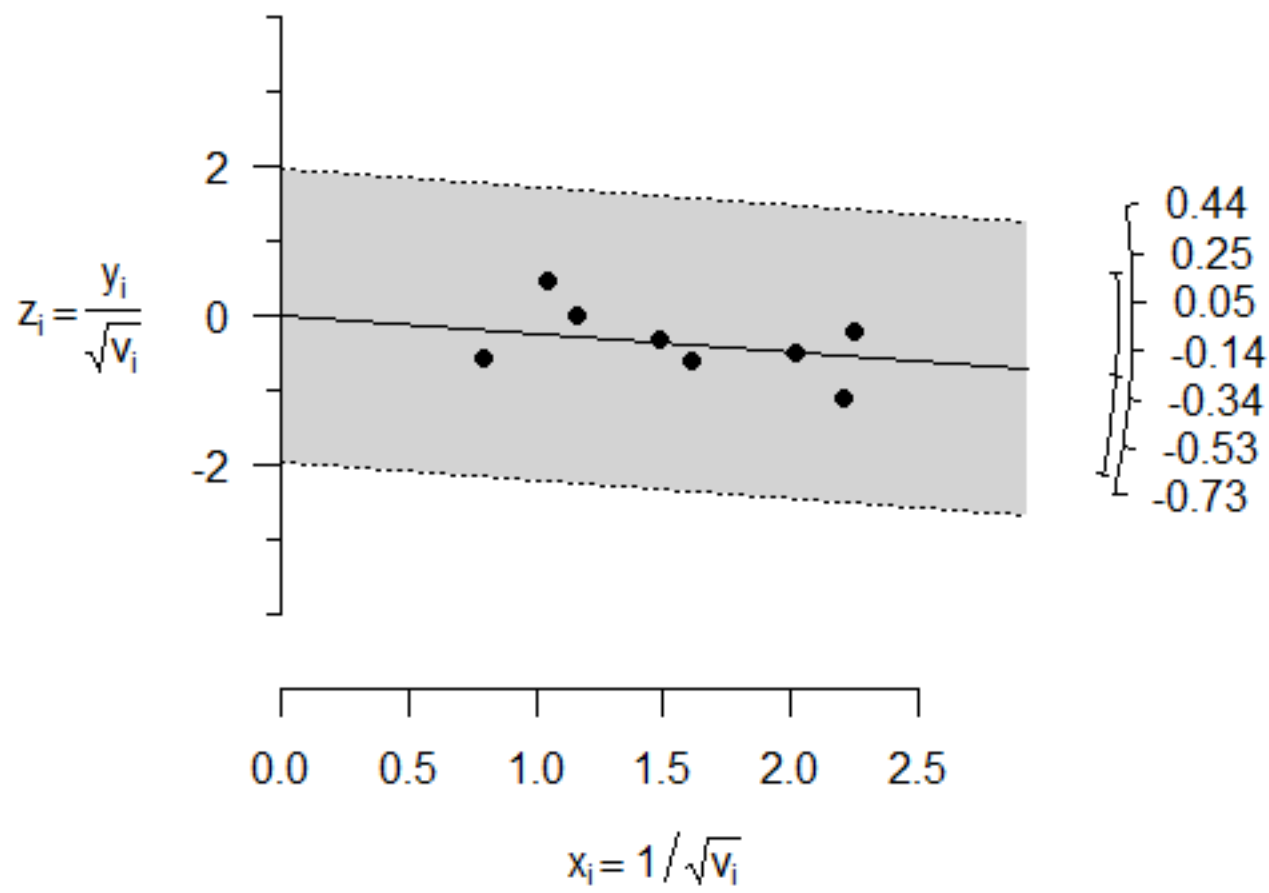

Supplement: S12 Fig — (PDF) [file pone.0167670.s013.pdf]

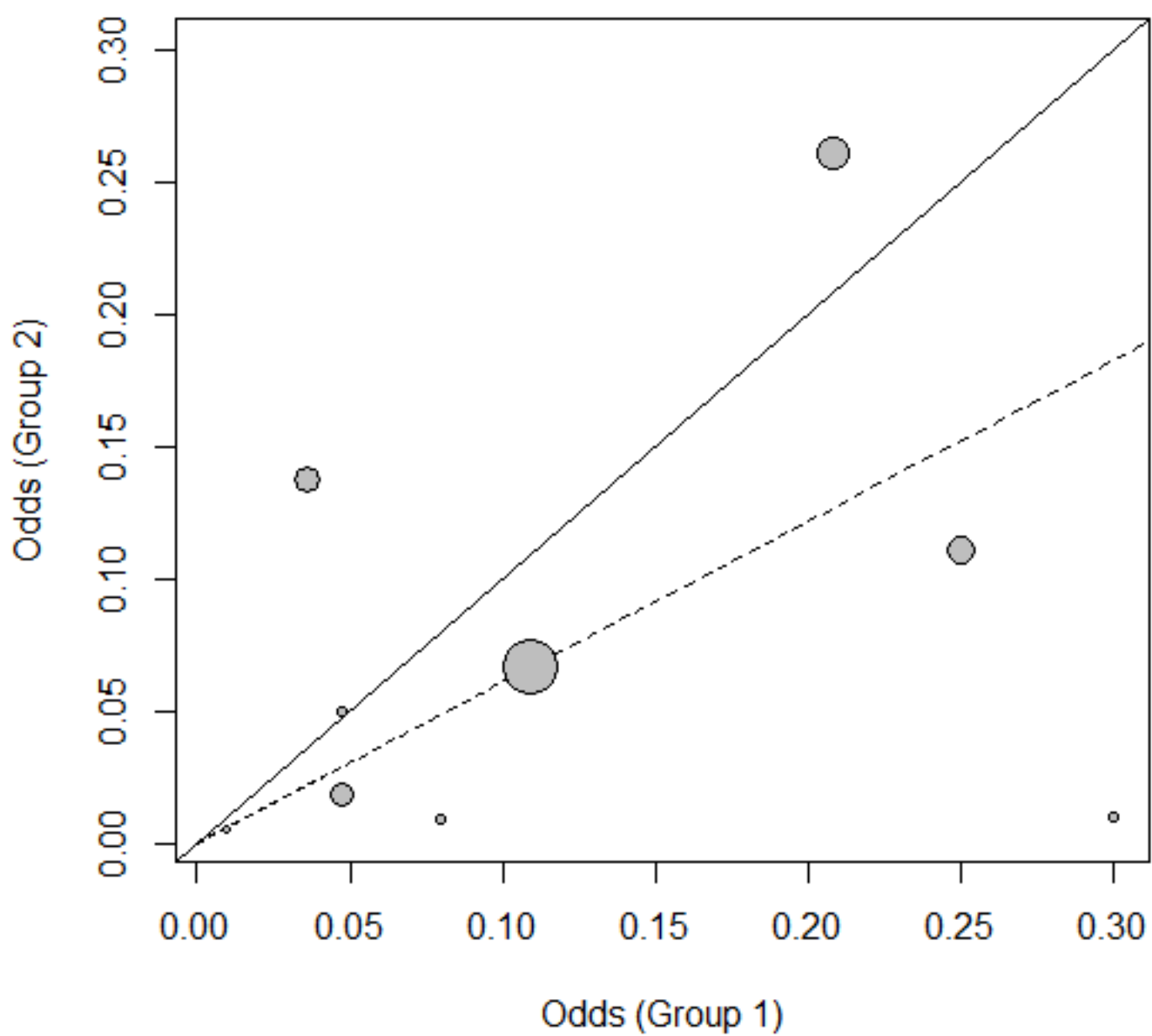

Supplement: S13 Fig — (PDF) [file pone.0167670.s014.pdf]

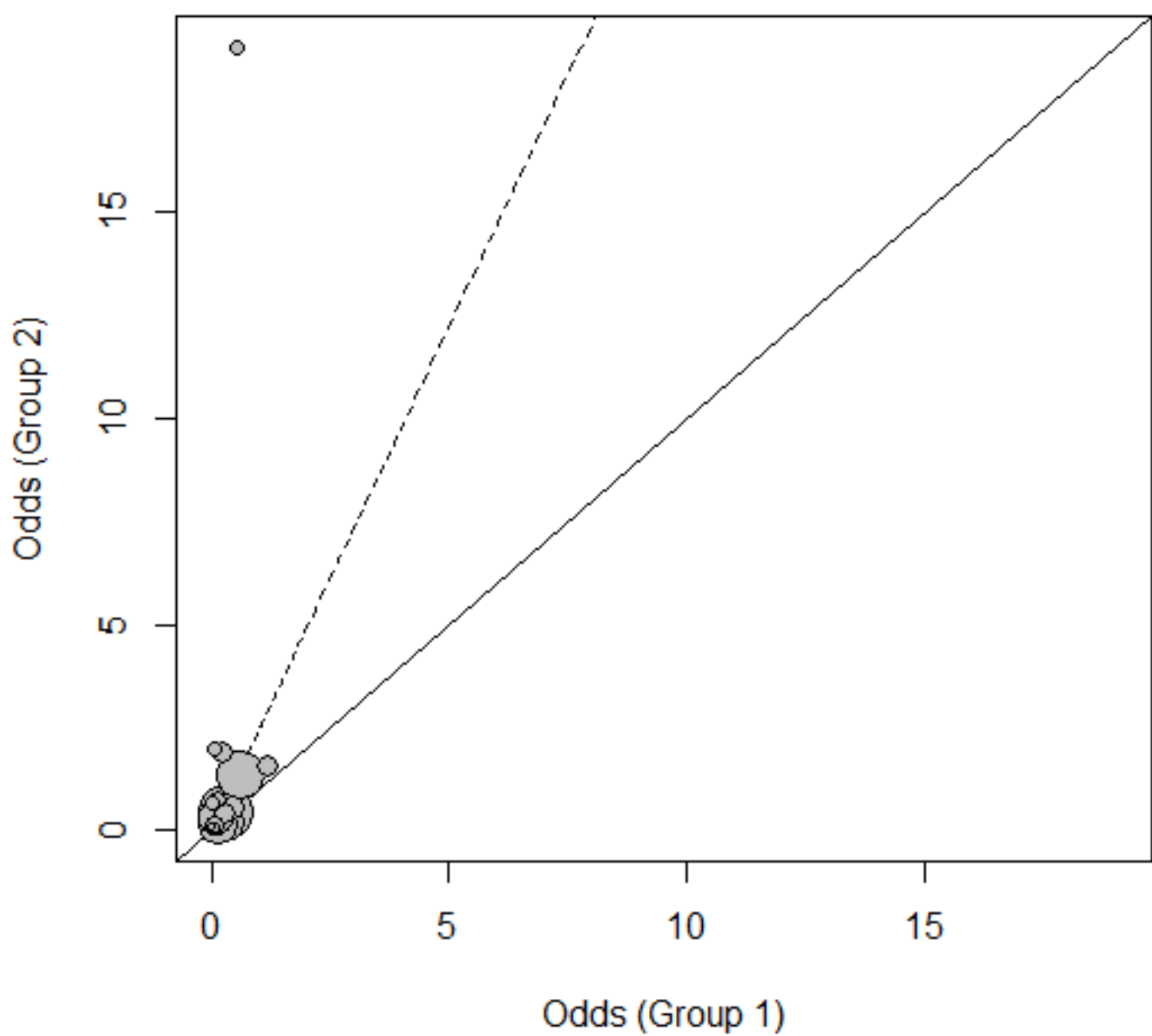

Supplement: S14 Fig — (PDF) [file pone.0167670.s015.pdf]

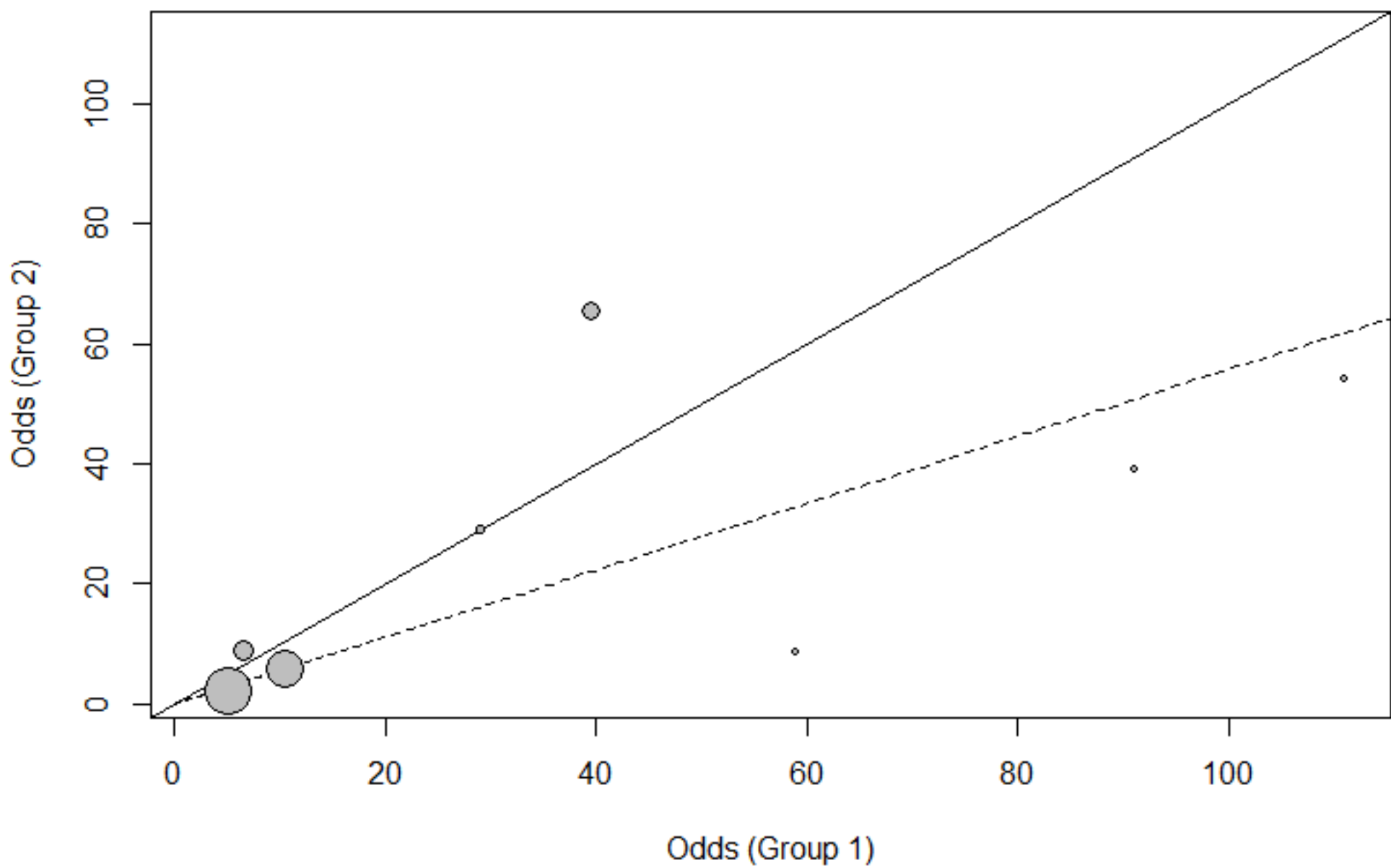

Supplement: S15 Fig — (PDF) [file pone.0167670.s016.pdf]

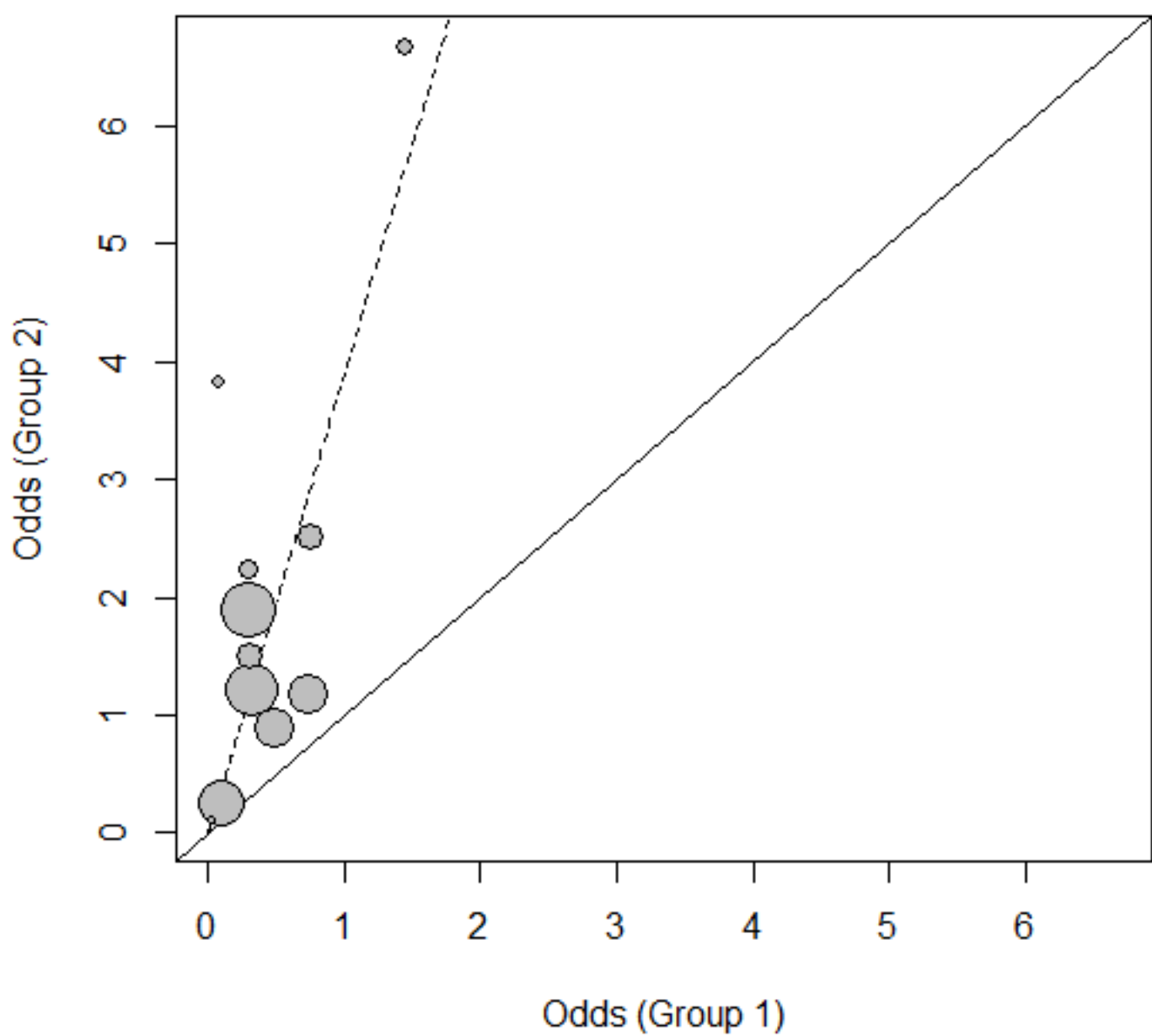

Supplement: S16 Fig — (PDF) [file pone.0167670.s017.pdf]

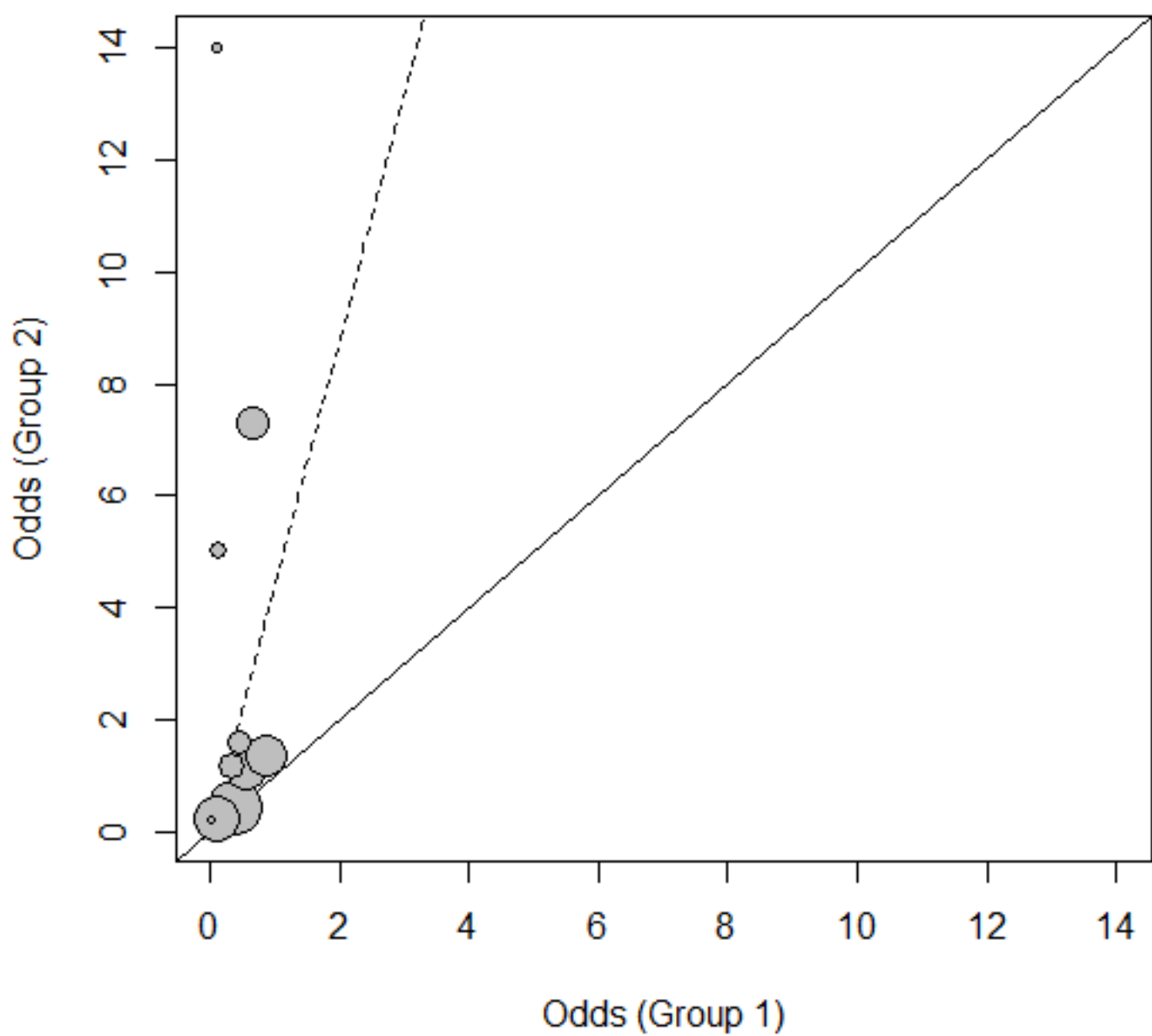

Supplement: S17 Fig — (PDF) [file pone.0167670.s018.pdf]

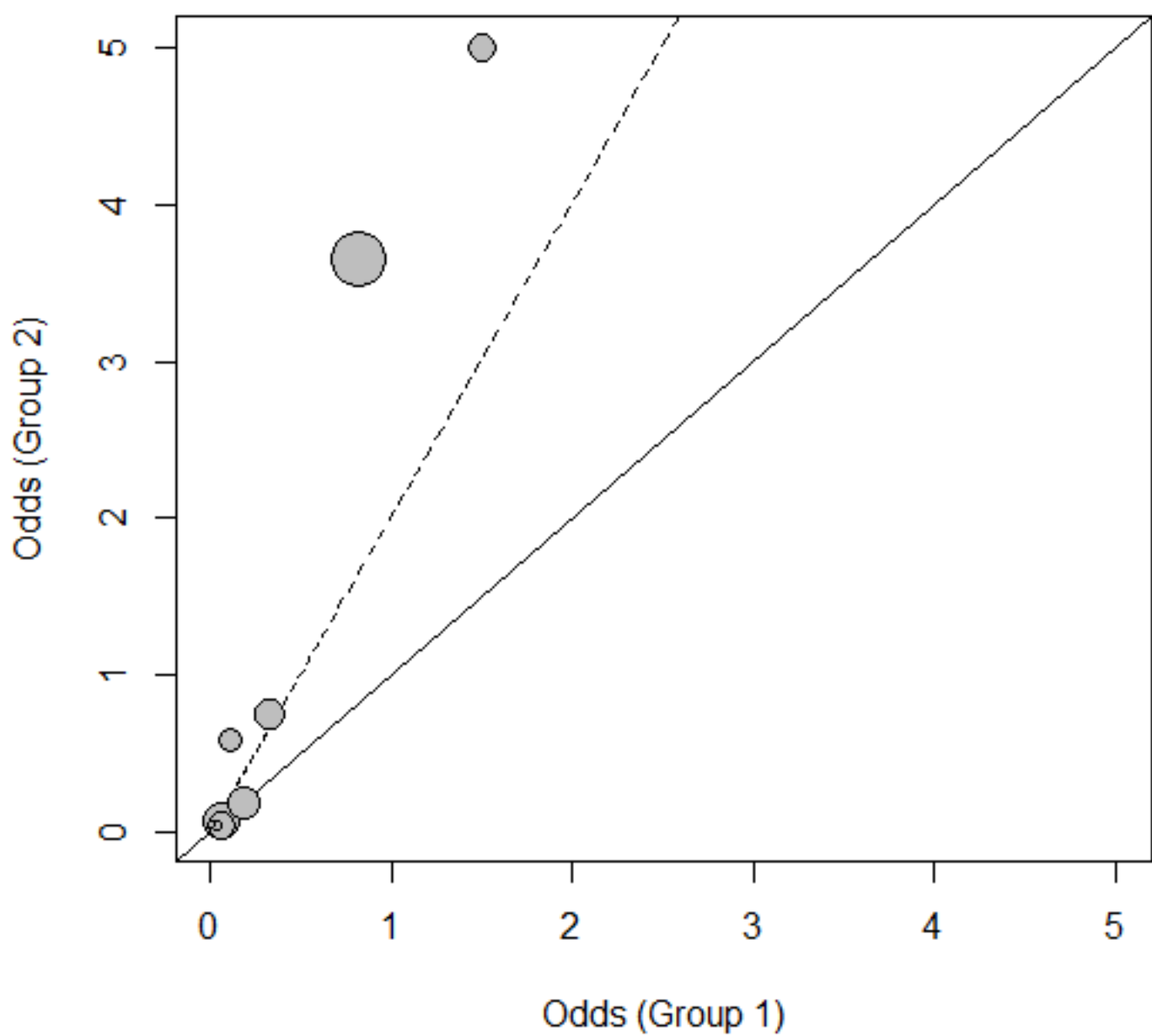

Supplement: S18 Fig — (PDF) [file pone.0167670.s019.pdf]

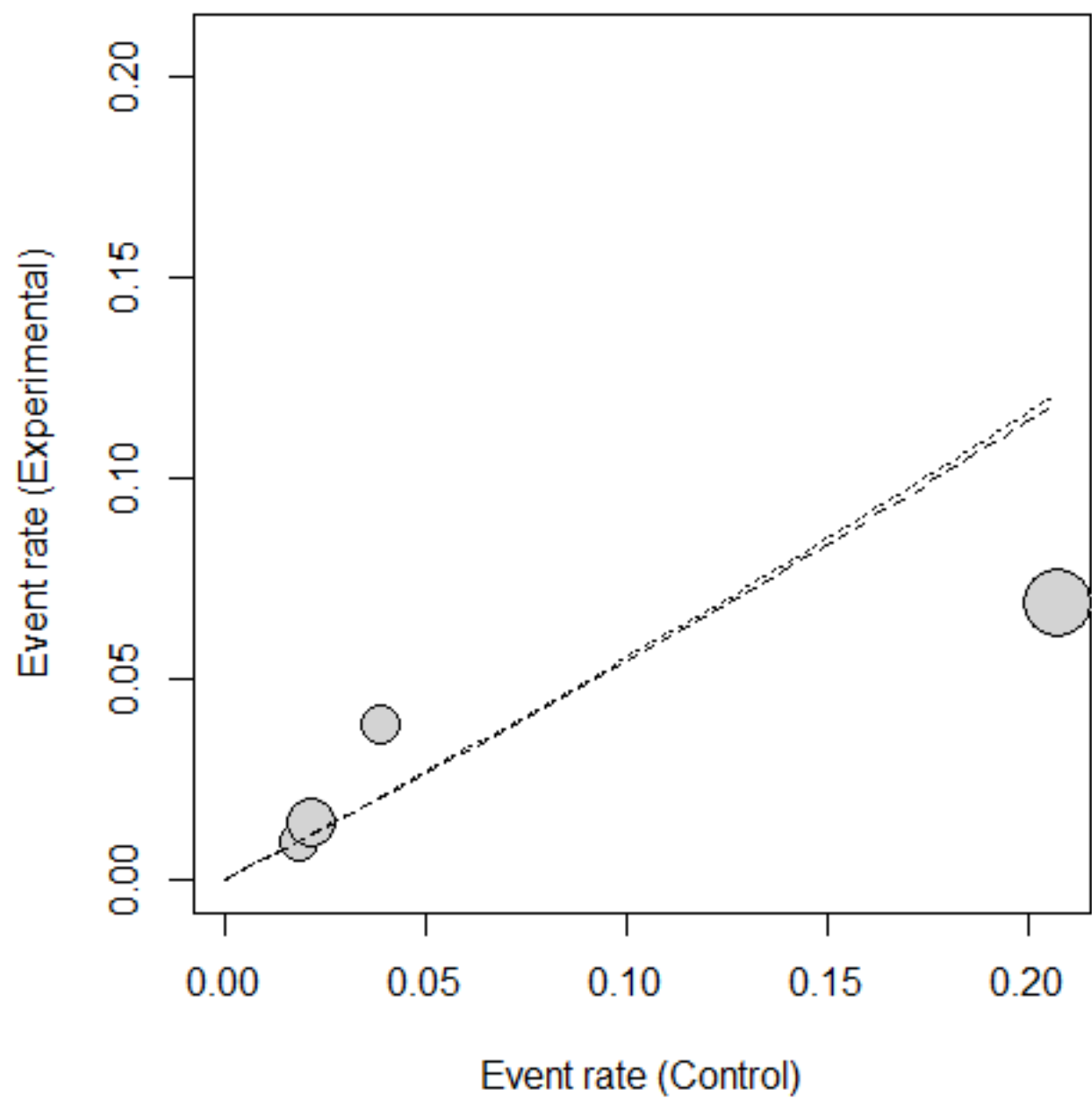

Supplement: S19 Fig — (PDF) [file pone.0167670.s020.pdf]

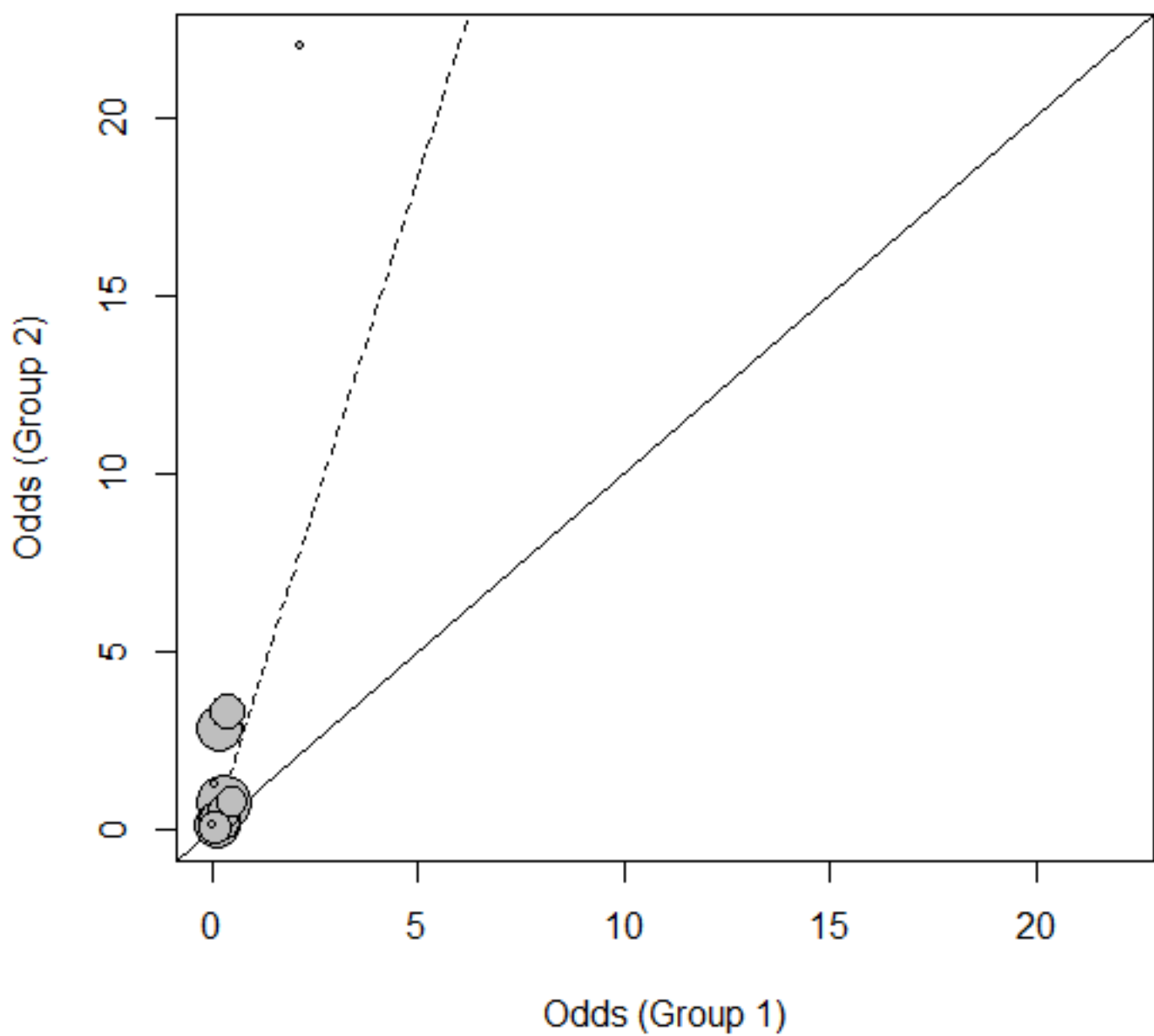

Supplement: S20 Fig — (PDF) [file pone.0167670.s021.pdf]

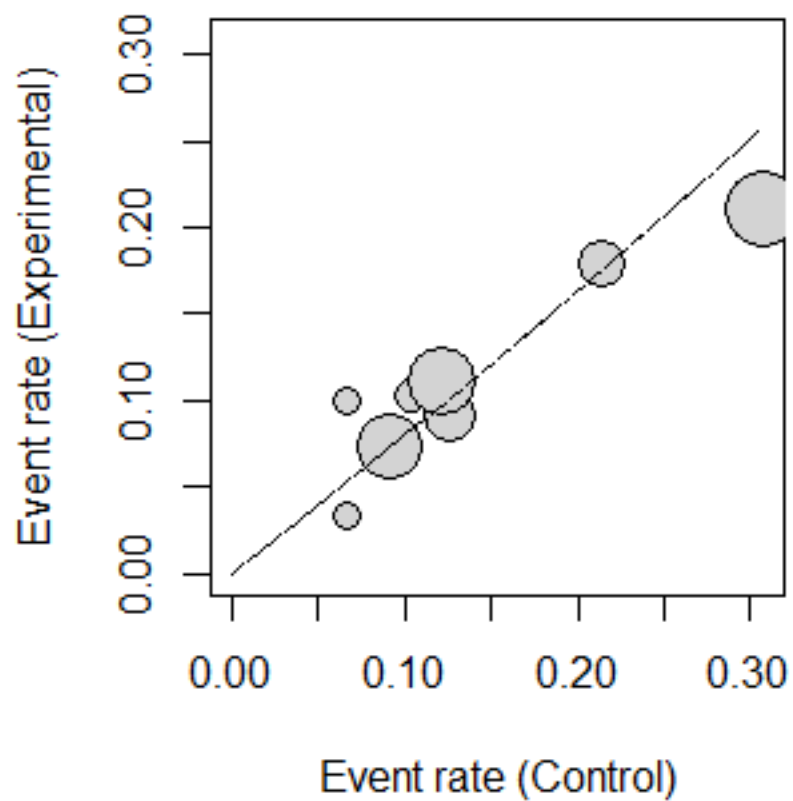

Supplement: S21 Fig — (PDF) [file pone.0167670.s022.pdf]

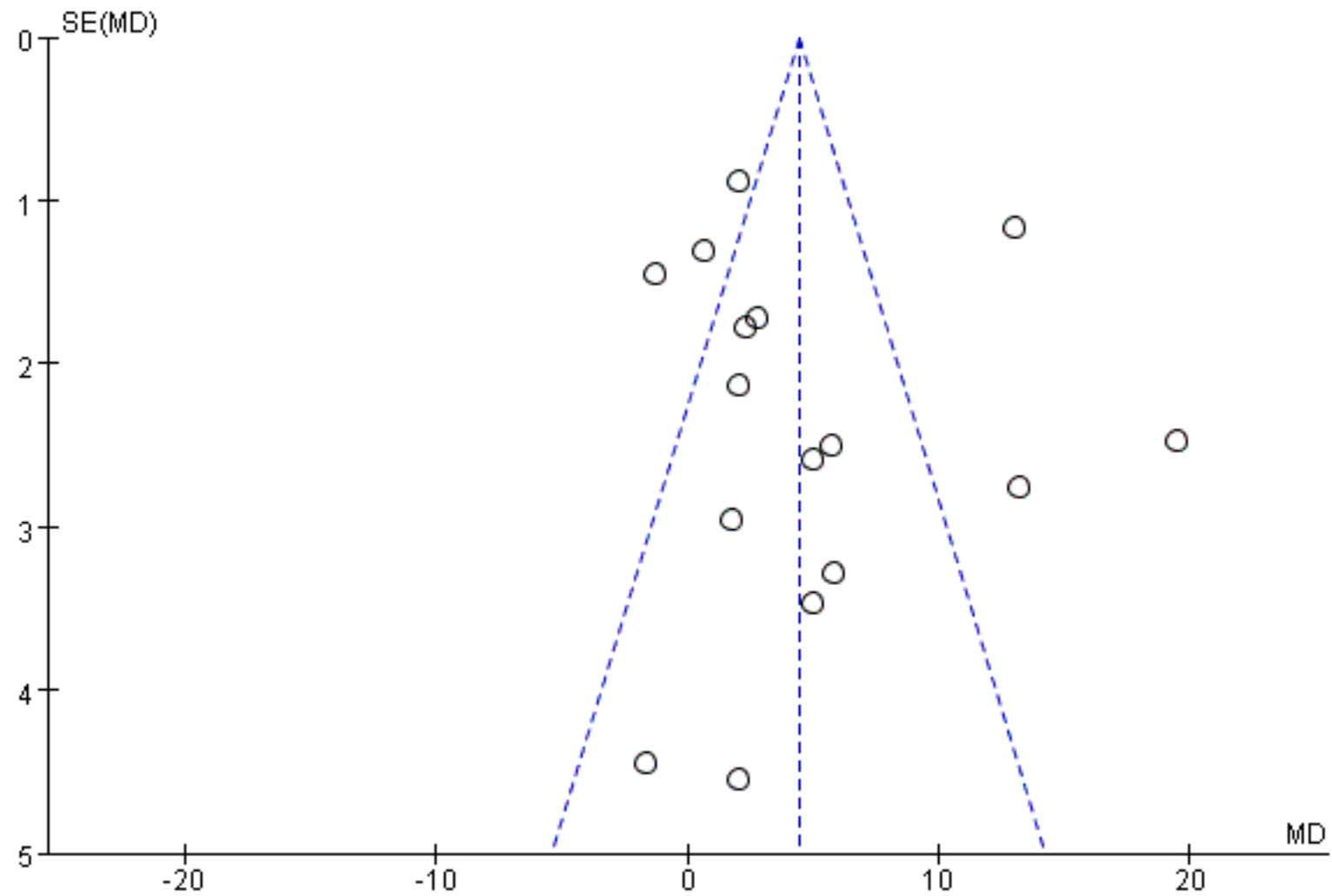

Supplement: S22 Fig — (PDF) [file pone.0167670.s023.pdf]

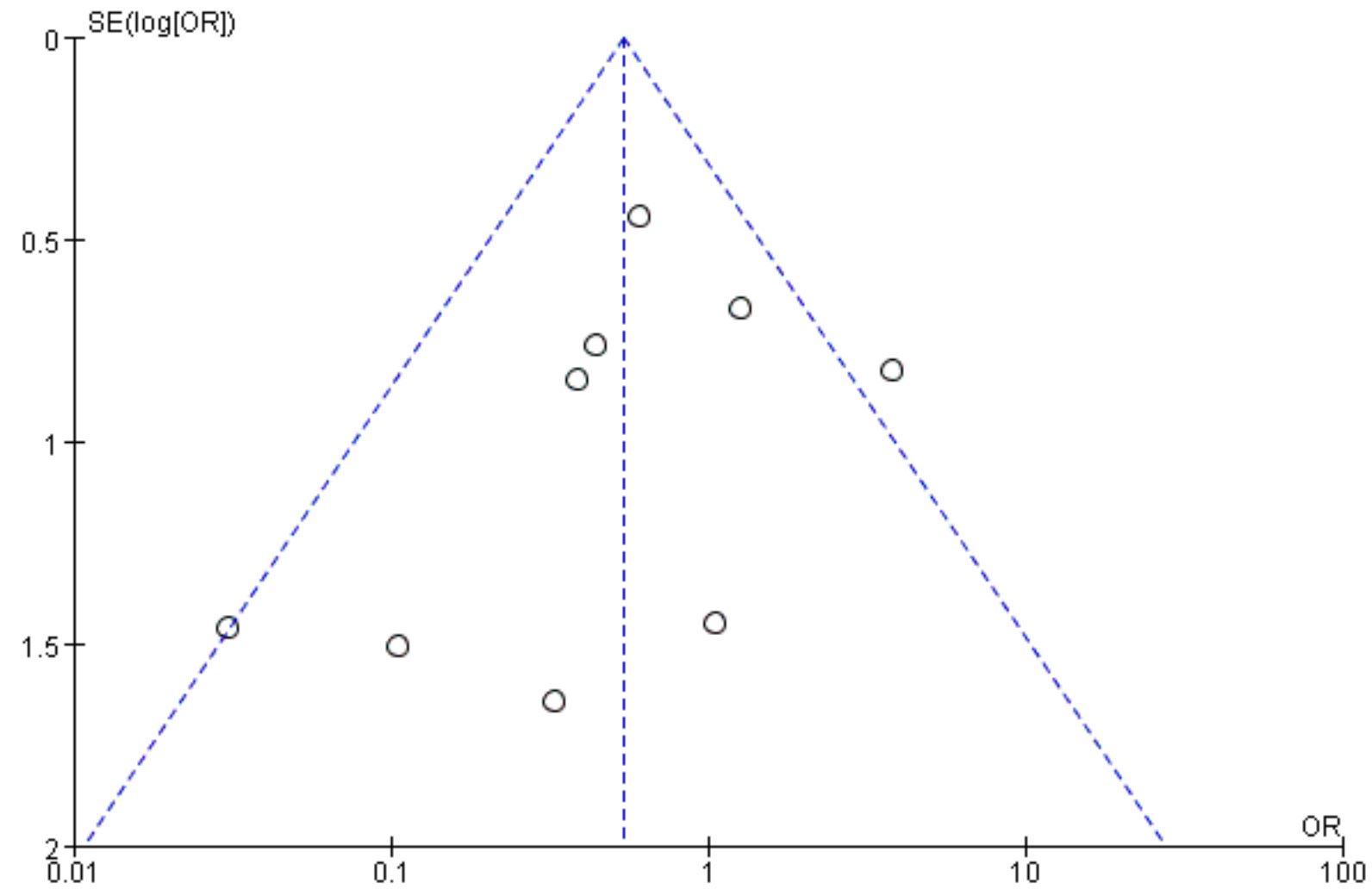

Supplement: S23 Fig — (PDF) [file pone.0167670.s024.pdf]

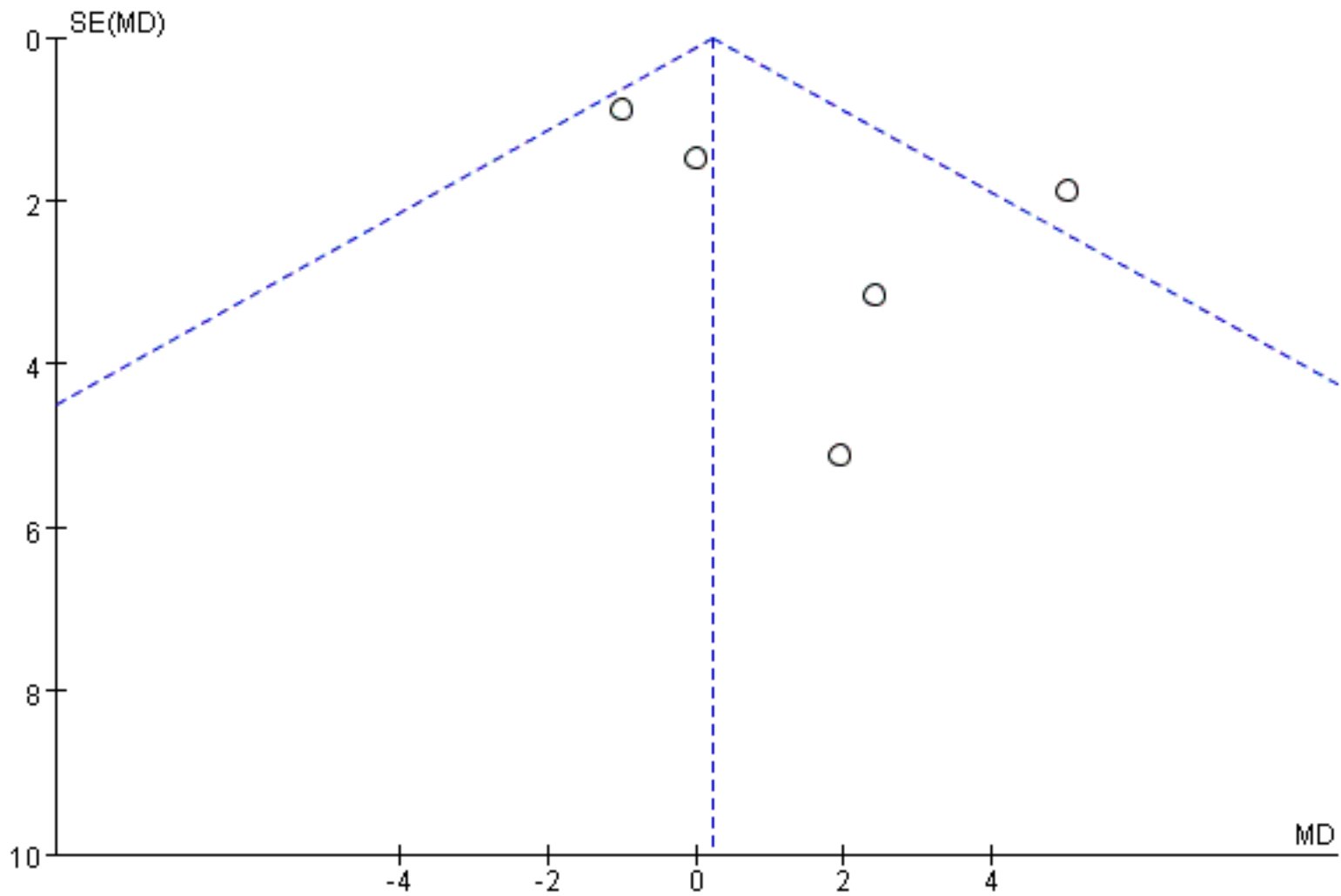

Supplement: S24 Fig — (PDF) [file pone.0167670.s025.pdf]

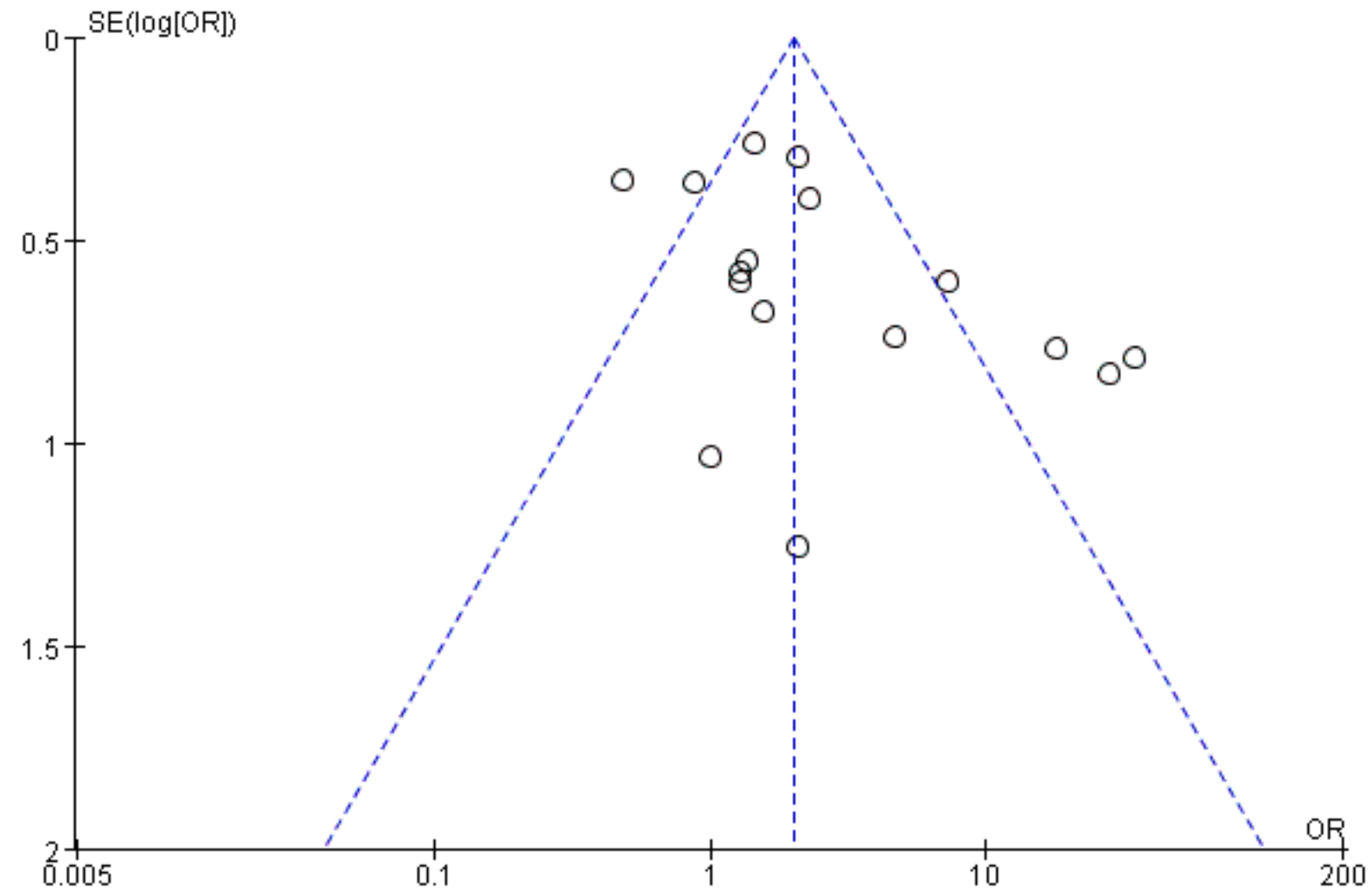

Supplement: S25 Fig — (PDF) [file pone.0167670.s026.pdf]

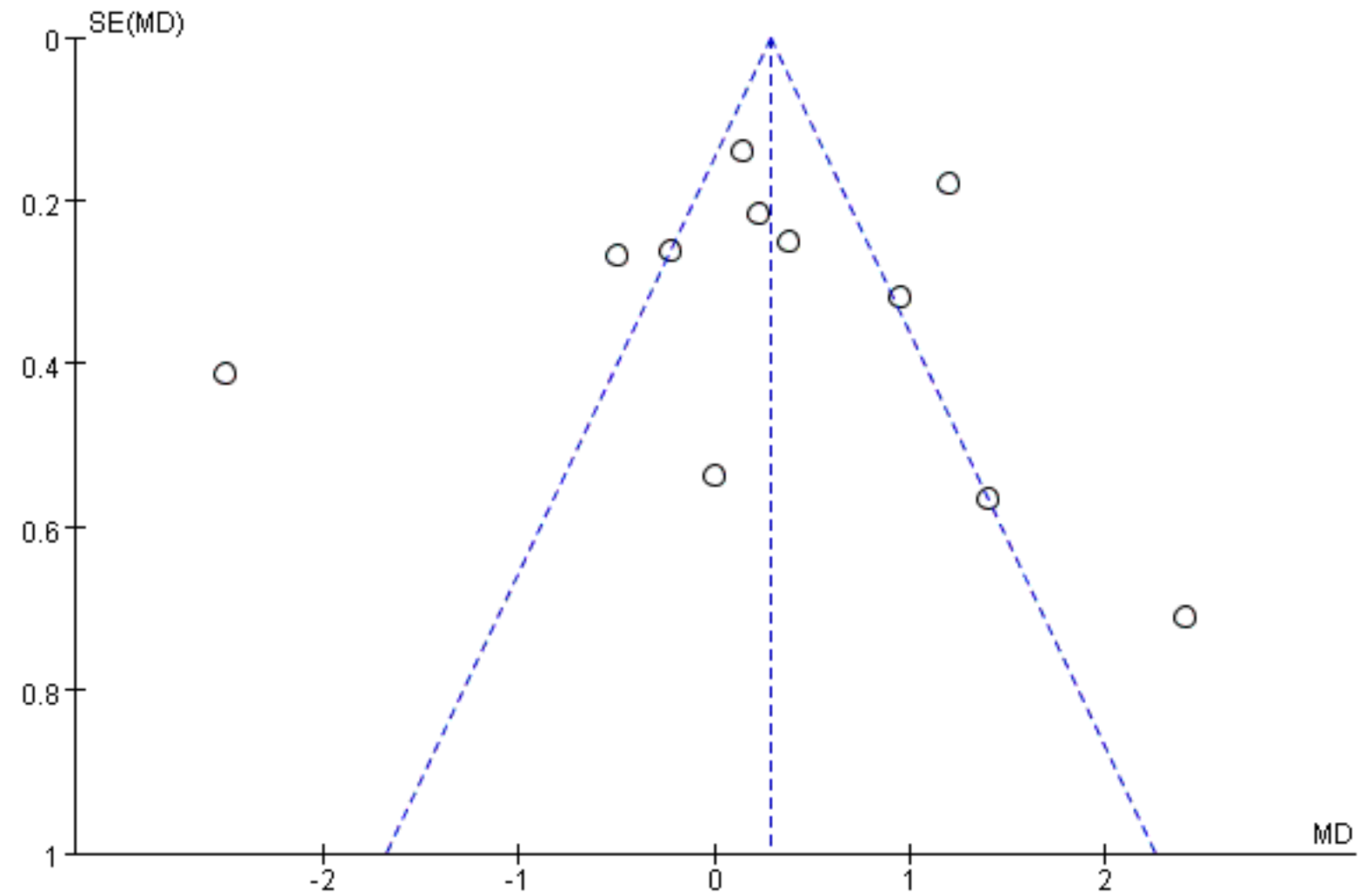

Supplement: S26 Fig — (PDF) [file pone.0167670.s027.pdf]

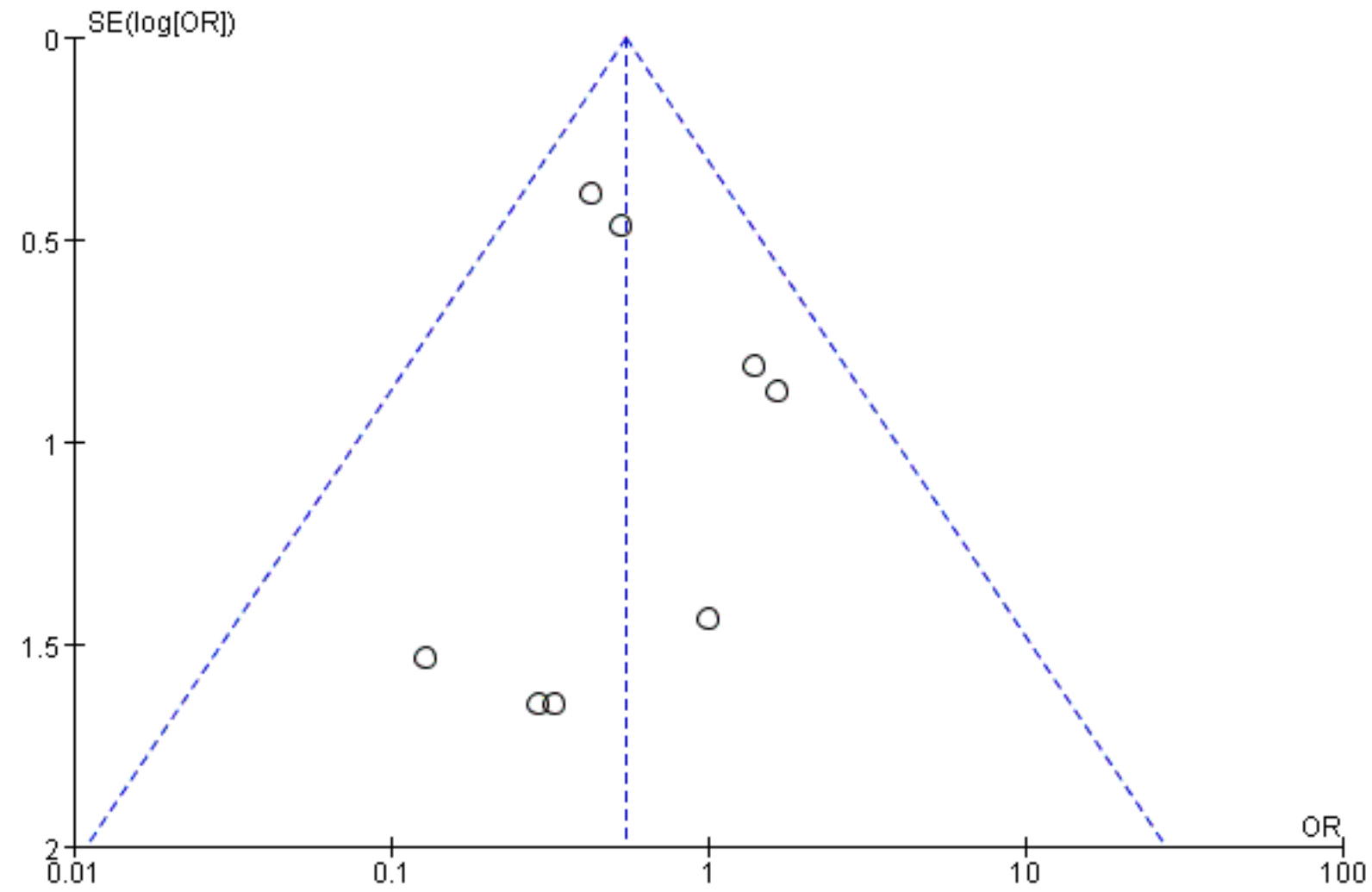

Supplement: S27 Fig — (PDF) [file pone.0167670.s028.pdf]

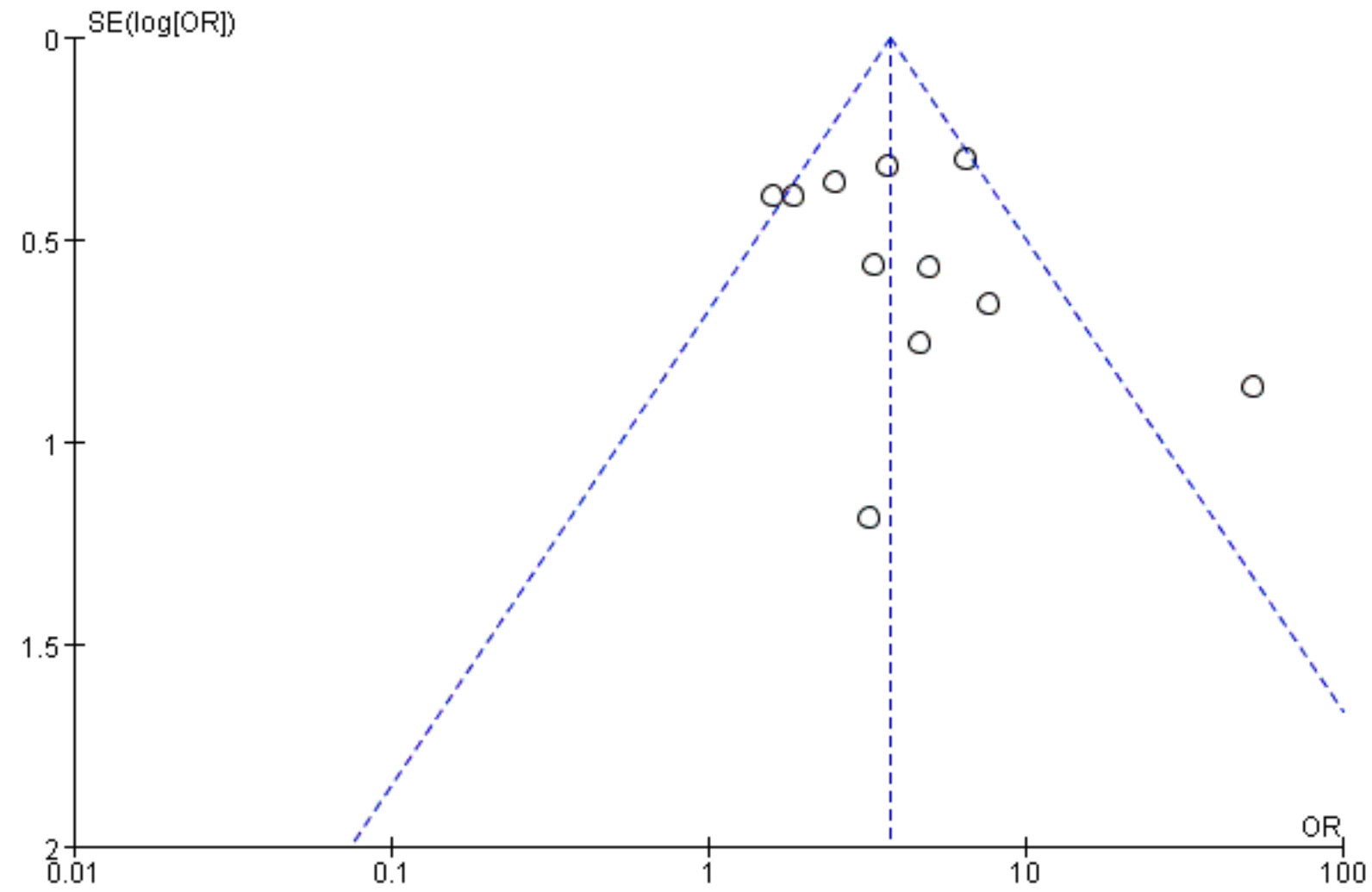

Supplement: S28 Fig — (PDF) [file pone.0167670.s029.pdf]

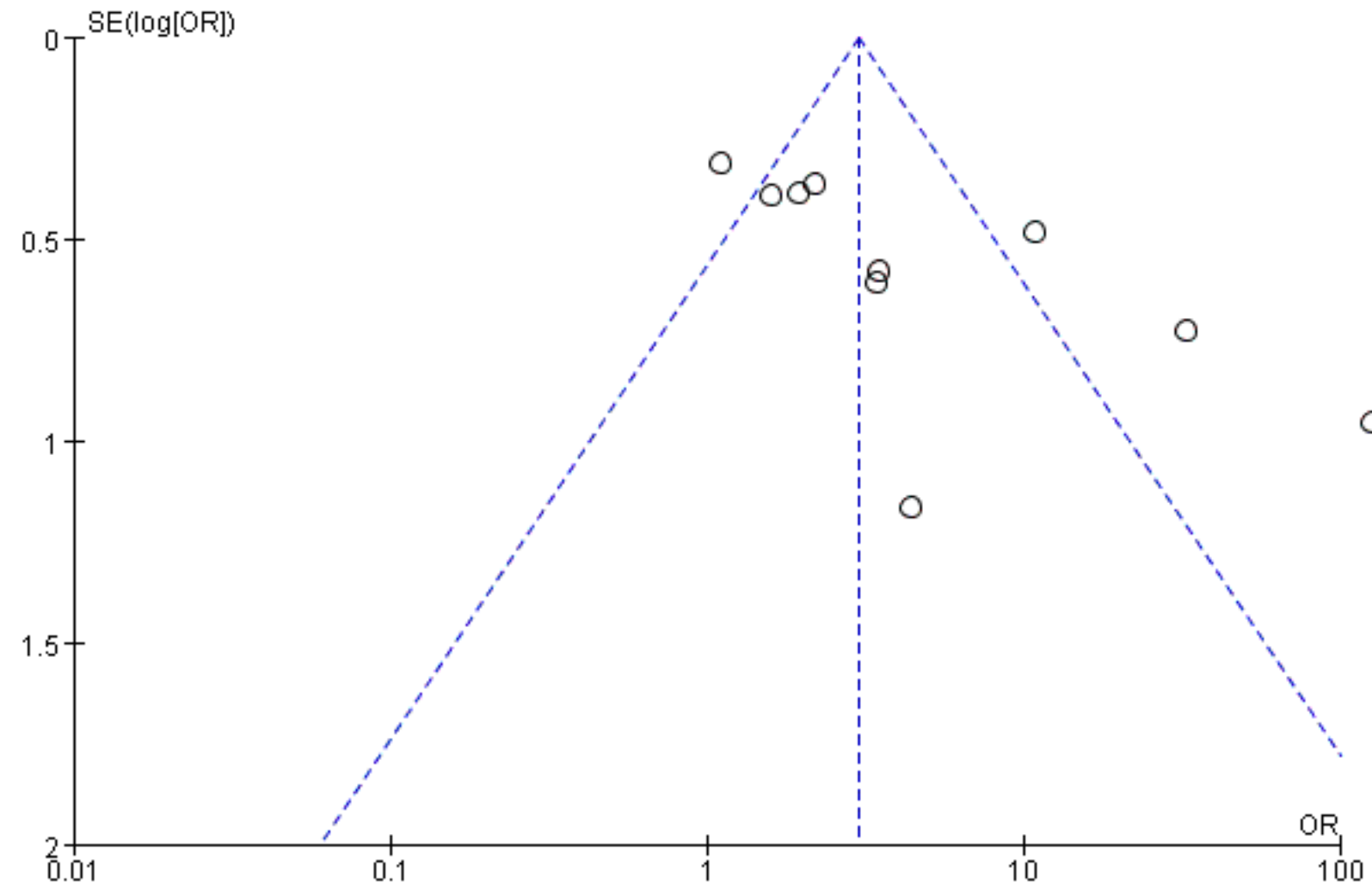

Supplement: S29 Fig — (PDF) [file pone.0167670.s030.pdf]

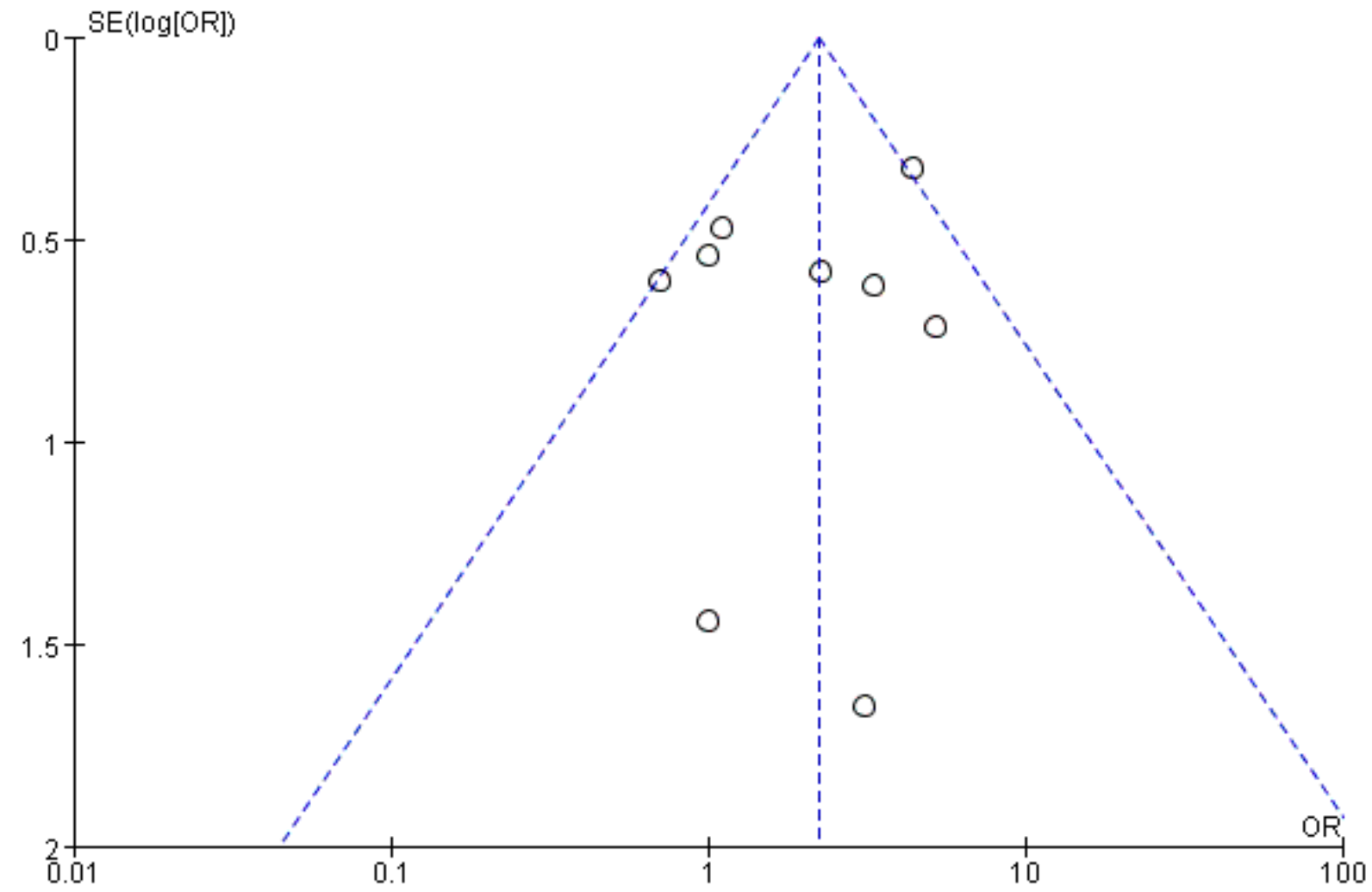

Supplement: S30 Fig — (PDF) [file pone.0167670.s031.pdf]

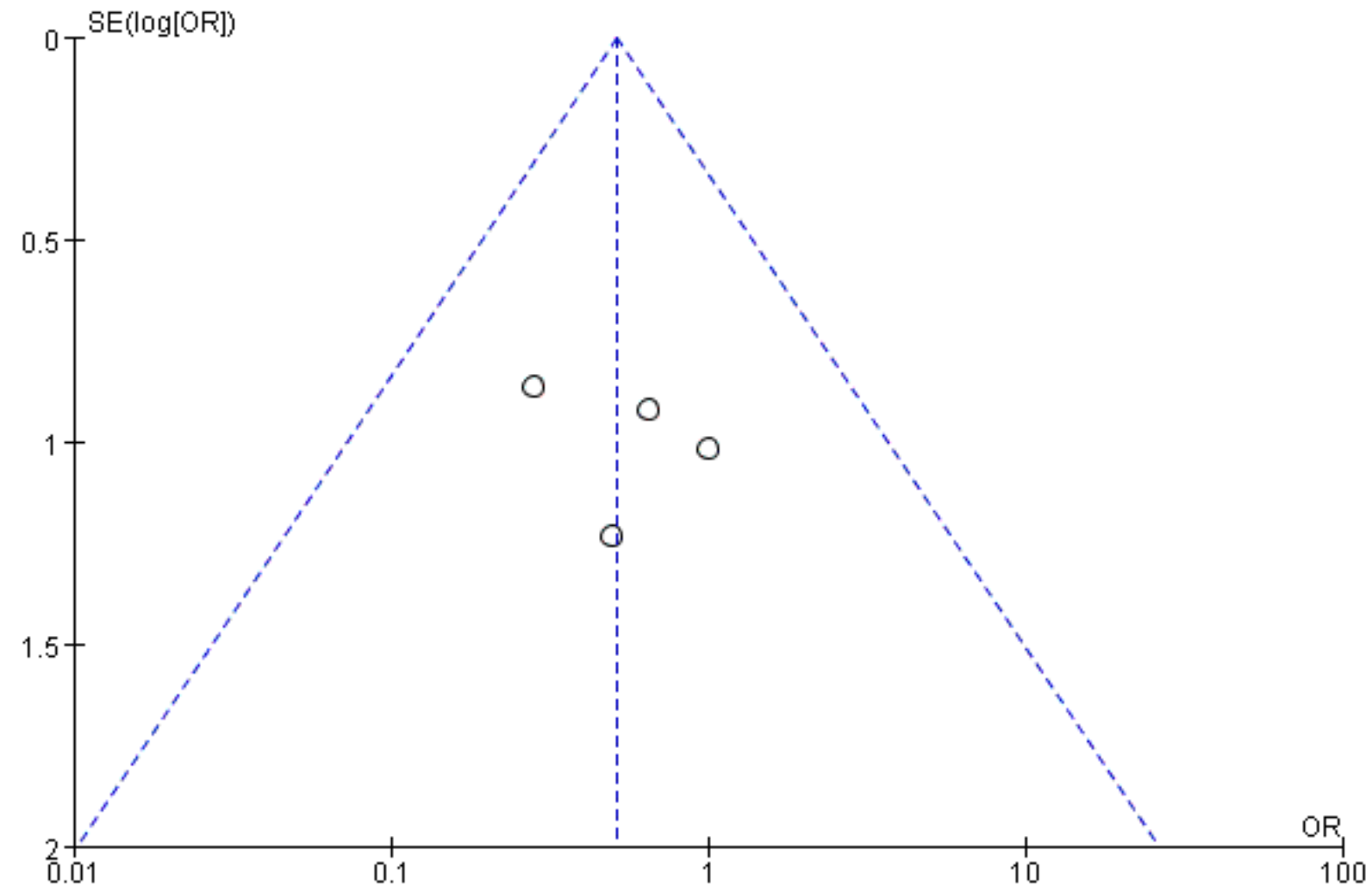

Supplement: S31 Fig — (PDF) [file pone.0167670.s032.pdf]

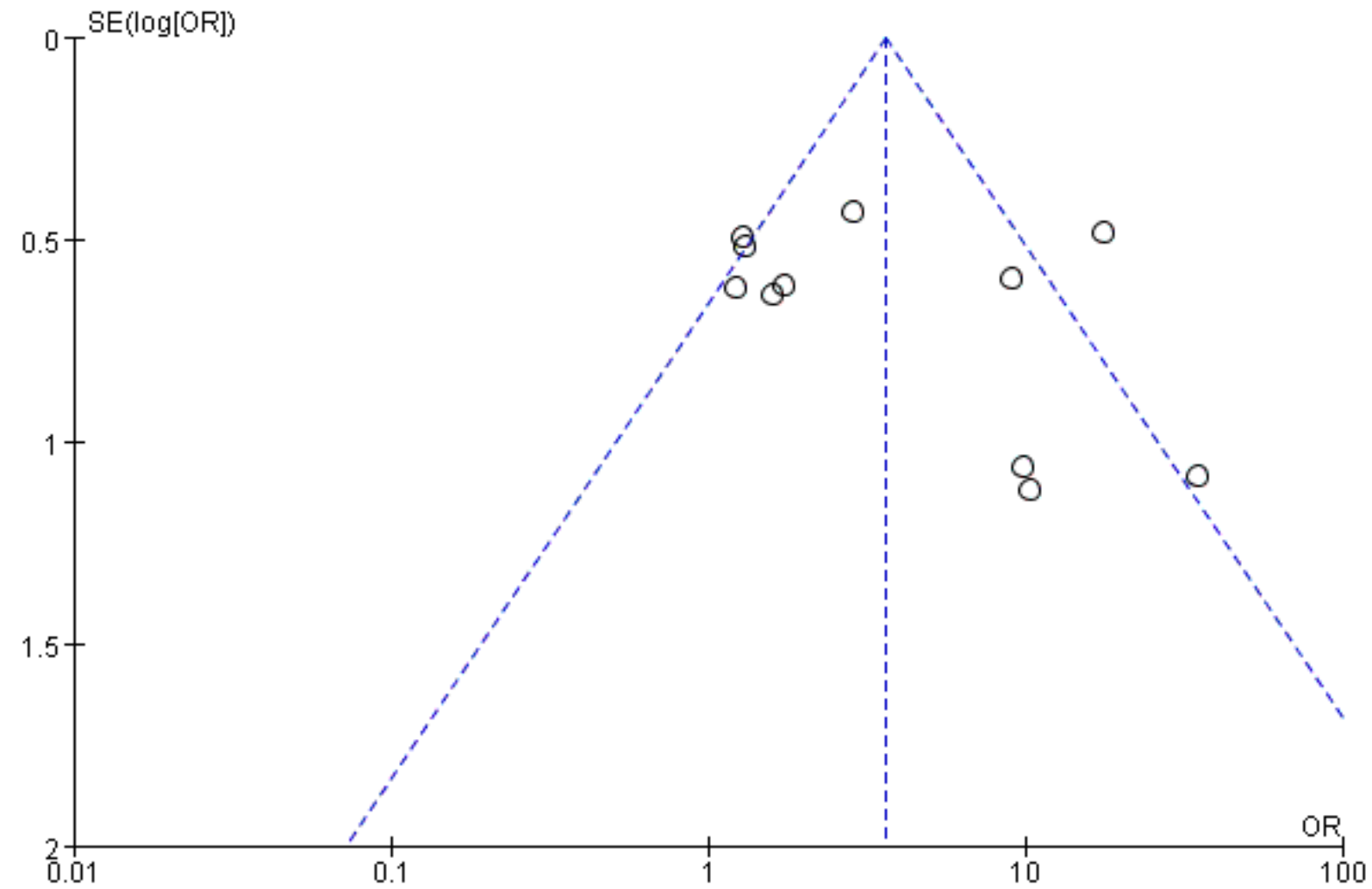

Supplement: S32 Fig — (PDF) [file pone.0167670.s033.pdf]

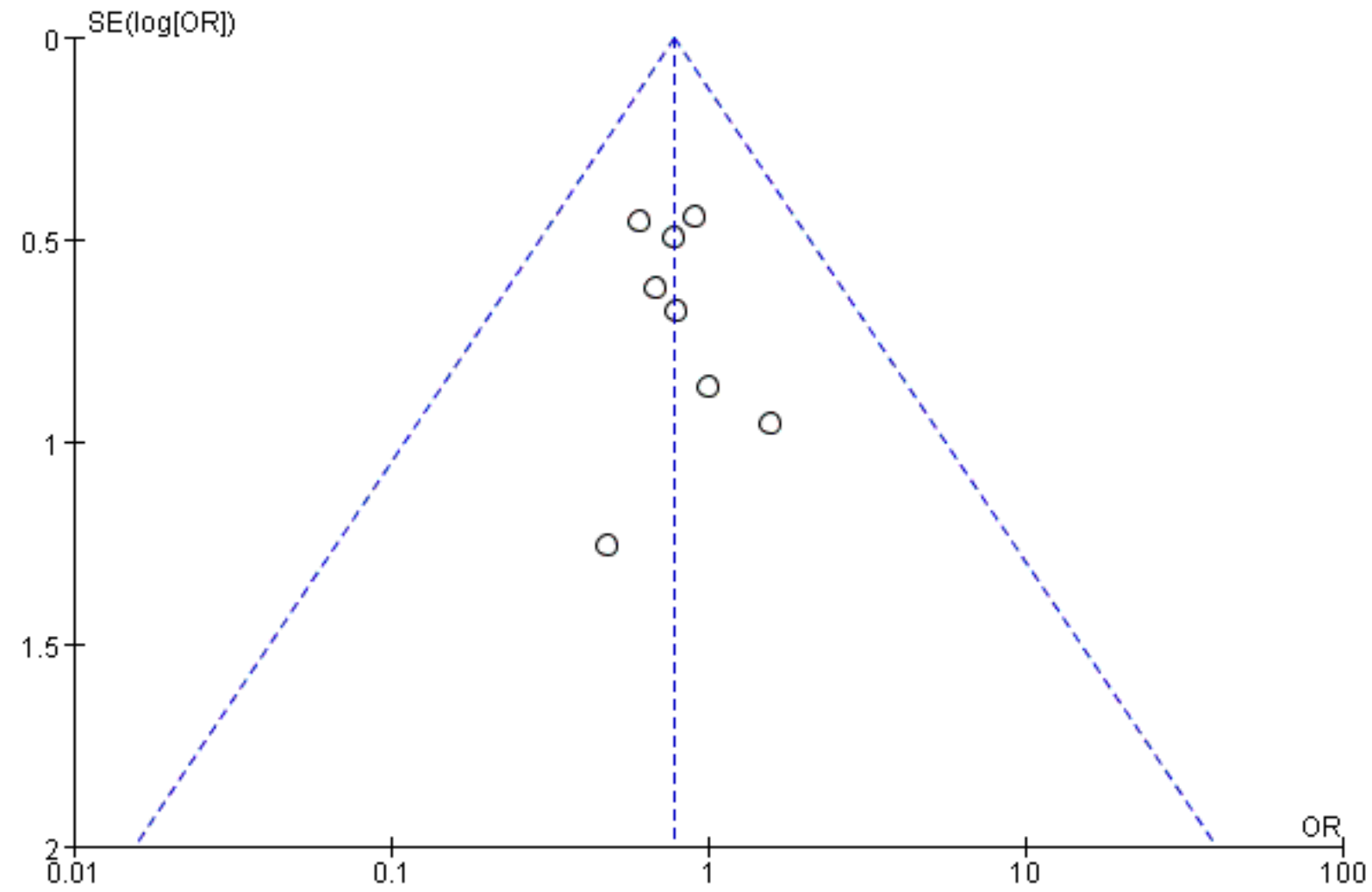

Supplement: S33 Fig — (PDF) [file pone.0167670.s034.pdf]
